# Metabolic and Transcriptomic Profiling of *Lilium* Leaves Infected With *Botrytis elliptica* Reveals Different Stages of Plant Defense Mechanisms

**DOI:** 10.3389/fpls.2021.730620

**Published:** 2021-09-22

**Authors:** Nan Chai, Jie Xu, Rumeng Zuo, Zhengqiong Sun, Yulin Cheng, Shunzhao Sui, Mingyang Li, Daofeng Liu

**Affiliations:** ^1^Chongqing Engineering Research Center for Floriculture, Key Laboratory of Horticulture Science for Southern Mountainous Regions of Ministry of Education, College of Horticulture and Landscape Architecture, Southwest University, Chongqing, China; ^2^Key Laboratory of Plant Hormones and Development Regulation of Chongqing, School of Life Sciences, Chongqing University, Chongqing, China

**Keywords:** *Lilium*, *Botrytis elliptica*, gray mold, phenylpropanoid pathway, flavonoid pathway, transcriptome, metabolic profiling

## Abstract

*Botrytis elliptica*, the causal agent of gray mold disease, poses a major threat to commercial *Lilium* production, limiting its ornamental value and yield. The molecular and metabolic regulation mechanisms of *Lilium*'s defense response to *B. elliptica* infection have not been completely elucidated. Here, we performed transcriptomic and metabolomic analyses of *B. elliptica* resistant *Lilium* oriental hybrid “Sorbonne” to understand the molecular basis of gray mold disease resistance in gray mold disease. A total of 115 differentially accumulated metabolites (DAMs) were detected by comparing the different temporal stages of pathogen infection. Kyoto Encyclopedia of Genes and Genomes (KEGG) enrichment analysis showed the differentially expressed genes (DEGs) and DAMs were enriched in the phenylpropanoid and flavonoid pathways at all stages of infection, demonstrating the prominence of these pathways in the defense response of “Sorbonne” to *B. elliptica*. Network analysis revealed high interconnectivity of the induced defense response. Furthermore, time-course analysis of the transcriptome and a weighted gene coexpression network analysis (WGCNA) led to the identification of a number of hub genes at different stages, revealing that jasmonic acid (JA), salicylic acid (SA), brassinolide (BR), and calcium ions (Ca^2+^) play a crucial role in the response of “Sorbonne” to fungal infection. Our work provides a comprehensive perspective on the defense response of *Lilium* to *B. elliptica* infection, along with a potential transcriptional regulatory network underlying the defense response, thereby offering gene candidates for resistance breeding and metabolic engineering of *Lilium*.

## Introduction

*Lilium* is one of the most economically important genera of ornamental monocots, whose species are used worldwide as cut flowers, garden plants, and potted plants. However, both the ornamental value and yield of commercial *Lilium* are often restricted by gray mold (Cui et al., 2018b). *Lilium* is highly susceptible to gray mold disease, and its effect is compounded by high humidity and low temperature (Hsieh et al., 2001). Gray mold disease, also known as leaf blight disease, is caused by the necrotrophic pathogens *Botrytis cinerea* and *Botrytis elliptica*. Among these, *B. cinerea* has been widely studied as a model necrotrophic fungus, as it has a broad host range and can infect more than 200 plant species (Hsiang and Chastagner, 1991; Gonzalez et al., 2017), whereas *B. elliptica* has a narrow host range and especially infects *Lilium* (Huang et al., 2001; Van Baarlen et al., 2004). At the early stages of gray mold infection, hygrophanous lesions appear on *Lilium* leaves in the form of oval or circular spots that change in color from yellowish brown to reddish brown over time. Then, the disease spreads rapidly throughout the whole plant, and its control becomes difficult (Ma et al., 2018). Development of disease resistant *Lilium* cultivars is currently the most economical and effective way to prevent gray mold disease incidence and spread. Thus, understanding the defense mechanism of *Lilium* against *B. elliptica* will help accelerate the process of breeding gray mold resistance traits in *Lilium* (Peng et al., 2017; Liu et al., 2020).

To defend against pathogen attack, plants have evolved two layers of immunity: pathogen-associated molecular pattern (PAMP)-triggered immunity (PTI) and effector-triggered immunity (ETI) (Liu et al., 2020). pathogen-associated molecular patterns trigger PTI and confer basic resistance to the attacked plant, while resistance (*R*) genes, whose encoded products can specifically recognize the cognate effector or pathogen avirulence proteins, function to regulate ETI (Zhao et al., 2021). Both PTI and ETI induce the production of defense related secondary metabolites, pathogen-related transcription factors (TFs), and pathogenesis-related (PR) proteins and activate hormone signal transduction as well as calcium (Ca^2+^) signaling (Liu et al., 2020). Phenolic metabolites, being important components of the plant immune system, play crucial roles in how plants respond to various pathogenic infections. Phenylpropanoids are precursors of a wide range of phenolic compounds, such as flavonoids, isoflavonoids, and cumarins (Shetty et al., 2011). Increased accumulation of phenylalanine in plants via its exogenous treatment significantly reduces their susceptibility to pathogens (Martinez et al., 2017; Doppler et al., 2019; Kumar et al., 2020). Flavonoids another important type of phenolic metabolite, have been reported to engage in antibacterial activity and can inactivate cell envelope transport proteins and disrupt microbial membranes and the respiratory chain (Long et al., 2019). Some flavonoid metabolites have been to promote phytohormone signaling and strengthen host resistance to necrotrophic *B. cinerea* in *Arabidopsis thaliana* (Hong et al., 2015). Numerous defense-related genes, such as those encoding TFs and PR proteins, control these various immune responses (Kumar et al., 2016; Sun et al., 2020).

The interaction between lilies and gray mold has been investigated at the molecular level in a few studies. Cui et al. (2018b) identified 23 *LrWRKY* genes from the resistant species *Lilium regale*, and showed that the overexpression of *LrWRKY4* and *LrWRKY12* enhanced *B. cinerea* resistance in transgenic *Arabidopsis*. Several resistance genes and pathogen-related microRNAs (miRNAs) were identified in *B. elliptica*-infected *L. regale* through RNA-seq and miRNA-seq (Gao et al., 2017; Cui et al., 2018a). More recently, Fu et al. (2020) performed comparative RNA-seq analysis of the expression profiles of the monolignol pathway genes from *L. regale* after its inoculation with *B. cinerea*, and were the first to report *CCoAOMT* as a potential molecular target in *Lilium*. Nevertheless, the molecular and metabolic regulatory mechanisms underlying the defense response of *Lilium* to *B. elliptica* remain largely unknown. Next-generation sequencing (NGS) has accelerated the pace of genetic studies by facilitating *de novo* genome assemblies from sequence reads obtained using the Illumina technology (Unamba et al., 2015; Mazumdar and Chattopadhyay, 2016; Almeida et al., 2018). Considering the large size and highly heterozygous nature of the *Lilium* genome, NGS is the most suitable approach for conducting molecular research on *Lilium* in the absence of a reference genome sequence. Additionally, untargeted metabolomic analysis is a newly developed method used for qualitative and quantitative analyses of various metabolites in plants (Abu-Nada et al., 2007; Parker et al., 2009; Lloyd et al., 2011). Combing transcriptome and metabolome investigations thus offers a feasible way to reliably reveal the various signals conveyed by *Lilium* after its infection with *B. elliptica*.

Here, we performed transcriptomic and metabolomic analyses of the *Lilium* oriental hybrid cultivar “Sorbonne”, which is known for its high volume of sales and strong resistance to gray mold (Zhang et al., 2015; Gao et al., 2018). The objectives of this study were to identify the major metabolic pathways that operate in *Lilium* after inoculation with *B. elliptica*, and to define a plausible transcriptional regulatory mechanism responsible for that response. The results of this study provide key insights into the transcriptional and metabolic mechanisms underlying the defense response of *Lilium* to *B. elliptica* infection.

## Materials and Methods

### Plant Cultivation and Pathogen Inoculation

The high resistant *Lilium* oriental hybrid cultivar “Sorbonne” (Gao et al., 2018), was used in this study. Bulbs were stored in a refrigerator (4°C) for 28 days, and then placed at the bottom of 15-cm deep pots filled with turf: vermiculite: perlite substrate (1:1:1, v/v/v). The pots containing the bulbs were placed in a greenhouse for 45 days at 25°C under 12-h light/12-h dark photoperiod, with photosynthetic photon flux density of 240 μmol·m.2·s-1 (Jang et al., 2018).

*Botrytis elliptica* strain 36423, isolated from symptomatic *Lilium* plants, was purchased from the Agricultural Cultural Collection of China (http://www.accc.org.cn/). The mycelium was cultured on potato dextrose agar (PDA, pH 5.8; Coolaber, China) medium in Petri dishes (9 cm diameter) at 25°C in the dark for 1 week.

Fully expanded, but not senescent, leaves were collected from *Lilium* plants at the flower bud stage, and inoculated with *B. elliptica*, according to the “detached leaves inoculation methods” of Gao et al. (2018). Before inoculation, all utensils and water were autoclaved at 121°C for 20 min, and the detached leaves were wiped clean using cotton soaked with sterile water. To inoculate the detached leaves, *B. elliptica* mycelium discs (5-mm diameter) were collected from the PDA plates using a sterilizing puncher, and then used to inoculate the abaxial surface of the detached lily leaves *in vitro*. Each leaf was inoculated with six mycelium discs (inoculated treatment), while those uninoculated with *B. elliptica* served as the control treatment. The inoculated and uninoculated leaves were placed in Petri dishes (15 cm diameter) lined with moist filter paper. The filter paper and cotton surrounding the leaf petiole were soaked with sterile water to maintain humidity within a range of 90–100%.

The detached leaves were sampled and photographed at 6, 8, 12, 24, 36, 48, and 72 h post inoculation (hpi). The area of each lesion was measured with the ImageJ software (https://imagej.nih.gov/ij/). Superoxide dismutase (SOD) activity was assayed by monitoring the photoreduction of nitro blue tetrazolium (NBT), as describe previously (Li, 2000). Based on the results of the SOD activity assay and the phenotype of disease lesions, leaves in the control, and inoculated treated treatments were sampled at 6, 24, and 48 hpi (hereafter referred to as control_6 h, control_24 h, and control_48 h, and inoculated_6 h, inoculated_24 h, and inoculated_48 h, respectively) for transcriptome and metabolome sequencing. Three biological replicates were performed for each treatment, with each replicate containing three technical repeats.

### Metabolomics

The leaf samples were freeze-dried under vacuum using the Scientz-100F lyophilizer (Scientz, China), and then crushed at 30 Hz for 1.5 min using a grinder (MM 400; Retsch, Germany) to obtain a fine powder. Then, 100 mg of each powdered sample was extracted in 0.6 ml of 70% methanol. The samples were stored at 4°C overnight, during which time they were vortexed six times to hasten the extraction. Then, each sample was centrifuged at 10 000 × *g* for 10 min. The supernatant was filtered through a microporous membrane (pore size: 0.22 μm), and stored in sample bottles for ultra-performance liquid chromatography-tandem mass spectrometry (UPLC-MS/MS) analysis.

To analyze the extracts, UPLC (Shim-pack UFLC SHIMADZU CBM30A; https://www.shimadzu.com.cn/) and MS/MS (Applied Biosystems 4500 QTRAP; http://www.appliedbiosystems.com.cn/) were performed using a C18 chromatographic column (Waters ACQUITY UPLC HSS T3 C18; 1.8 μm, 2.1 × 100 mm) with solvent A (0.04% acetic acid in ultrapure water) and solvent B (0.04% acetic acid in acetonitrile) as the mobile phase. A 4-μl aliquot of each sample was injected into the column, and eluted using the following gradient program: 0 min with 95% A and 5% B; 0–10 min with 95–5% A and 5–95% B; 10–11 min with 5% A and 95% B; 11–11.1 min with 5–95% A and 95–5% B; and 11.1–14 min, 95% A and 5% B. The flow rate and column temperature were maintained at 0.35 mL/min and 40°C, respectively. The mass spectrometer parameters were set as follows: temperature of electrospray ionization: 550°C; voltage: 5.5 kV; curtain gas: 30 psi; collision-activated dissociation: high. Each ion pair was scanned and detected based on optimized declustering potential and collision energy in the triple quadrupole system.

Qualitative and quantitative analyses of the metabolites were performed using the multiple reaction monitoring (MRM), KEGG compound database, and MetWare database. Metabolites were identified based on their molecular weight, Mass Spectrometry (MS^2^) fragments, MS^2^ fragments isotope distribution, and retention time (RT). Through the MetWare self-developed intelligent secondary spectrum matching method, the secondary spectrum and RT of the metabolites in the project samples are intelligently matched one by one with the MetWare database. The MS tolerance and MS^2^ tolerance are set to 2 and 5 ppm, respectively. The peak area integral of all the mass spectrum peaks was derived after obtaining the metabolic substance spectrum analysis data of different samples, followed by an integral correction performed for the mass spectrum peak of the same metabolite occurring in different samples (Fraga et al., 2010). Quality control (QC) samples, i.e., samples prepared from a mixture of sample extracts, were used to analyze the reproducibility of the instrument under the same treatment method.

The “MetaboAnalystR 1.0.1” package in the R computing platform v 3.5.0 (R Core Team, 2018, Austria) was used to statistically analyze the metabolomics data and to generate plots. Orthogonal partial least squares discriminant analysis (OPLS-DA) was conducted using MetaboAnalystR to identify differentially accumulated metabolites (DAMs), with Variable Importance in Projection (VIP) score ≥ 1 and absolute Log_2_fold change (FC) ≥ 1. Pathways with significantly regulated metabolites mapped to it were then subjected to metabolite set enrichment analysis; their respective statistical significance was determined using the hypergeometric test and its *p*-value.

### Transcriptomics

A total of 18 cDNA libraries were sequenced using the NEBNext® Ultra™ RNA Library Prep Kit for Illumina® (NEB; USA). Clean reads obtained from each cDNA library were assembled *de novo* into unigenes using the Trinity (2.6.6.) platform (Haas et al., 2013). Functional annotations of the unigenes were determined using NCBI non-redundant (NR) protein, UniProt, KEGG, Gene Ontology (GO), and Clusters of Orthologous Groups of proteins (COG) databases (Kanehisa et al., 2008). Transcription factors and gene coding sequence (CDS) were used ITAK web online (http://itak.feilab.net/cgi-bin/itak/index.cgi) and TransDecoder (5.3.0, https://github.com/TransDecoder) software, respectively. Differentially expressed genes (DEGs) were analyzed using the DESeq2 (v1.22.2) software package in R, and subjected to the Benjamini–Hochberg method for multiple hypothesis testing (i.e., |log2FC| ≥ 1, FDR [false discovery rate] <0.05) (Love et al., 2014, Varet et al., 2016). Heat maps were constructed using the R package “pheatmap” (v1.0.12) and TBtools software v0.66836 (Chen et al., 2020). Venn diagrams were generated using Venn v1.6. Weighted gene coexpression network analysis (WGCNA) was performed using the R package “WGCNA” (Zhang and Horvath, 2005), and visualized using Cytoscape v1.7.251 (https://cytoscape.org/index.html).

### Quantitative Real-Time PCR (qRT-PCR)

To validate the RNA-seq data, the expression of 10 defense-related DEGs, including four DEGs involved in plant–pathogen interactions (Cui et al., 2018a) and six DEGs involved in the phenylpropanoid and flavonoid biosynthesis pathways, was analyzed by qRT-PCR. The qRT-PCR was performed using the SsoFast EvaGreen Supermix (Bio-Red, USA) on the CFX96 Real-Time PCR Detection System (Bio-Rad, USA) under the following conditions: 95°C for 30 s, followed by 39 cycles of 95°C for 5 s and 57°C for 5 s, melt curve 65–95°C, increment 0.5°C for 5s. The specificity of the primers was verified based on the unimodality of the melt curve. *Actin (ACT)* and *elongation factor 1* (*EF1*) served as reference genes (Cui et al., 2018a). Three biological replicates were performed for each treatment, with each replicate containing three technical repeats. Data were analyzed using the 2^−ΔΔCt^ method (Livak and Schmittgen, 2001). Primers used for qRT-PCR are listed in Supplementary Table 1.

## Results

### *B. elliptica*-Induced Lesions and Altered SOD Activity in *Lilium* Hybrid “Sorbonne”

**Figure 1:**
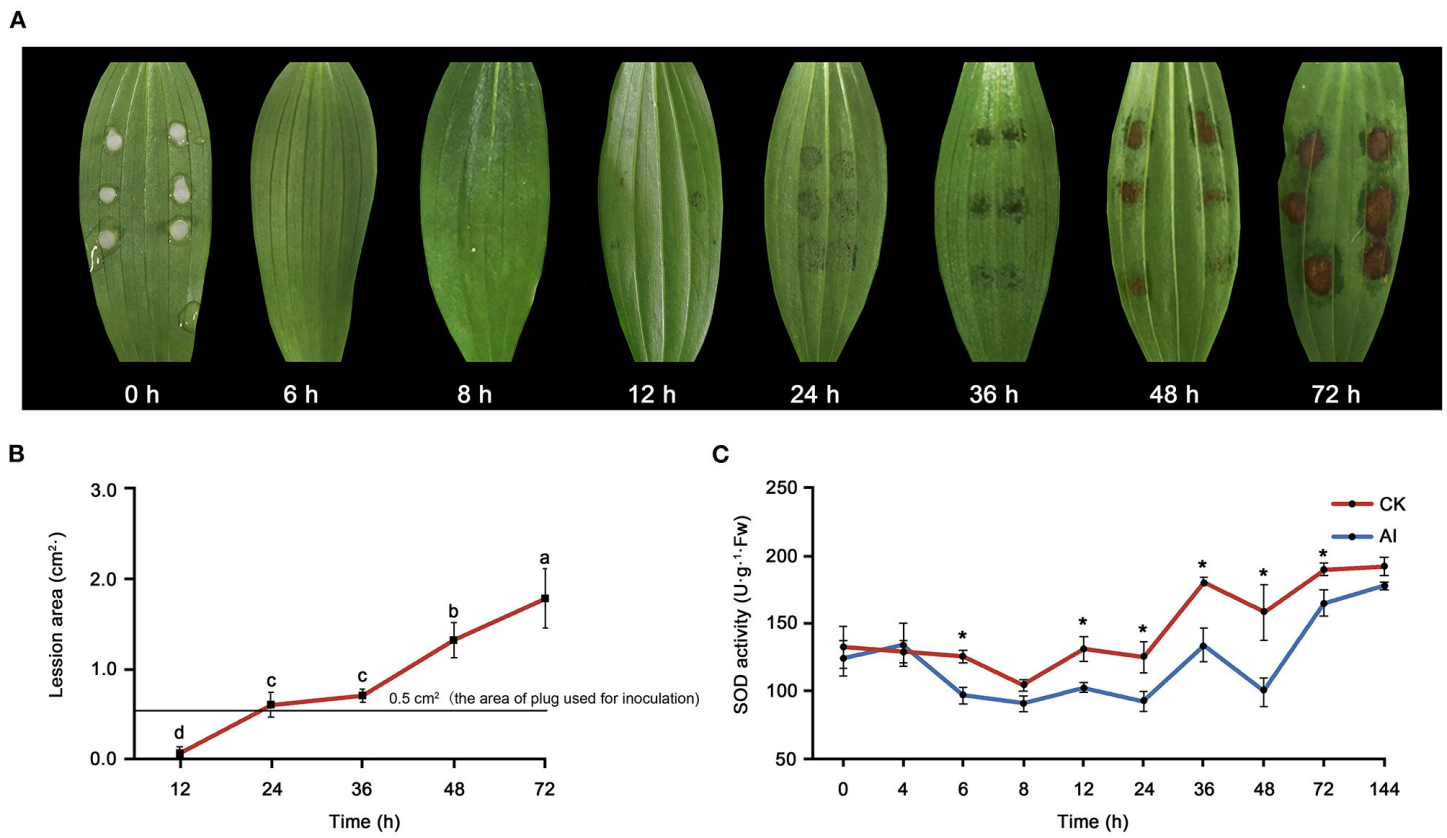
Evaluation of the response of *Lilium* oriental high resistant hybrid “Sorbonne” to *Botrytis elliptica* inoculation. **(A)** Progression over time (0–72 hpi) of symptoms on *B. elliptica* inoculated Sorbonne leaves. Each leaf is representative of nine repetitions. **(B)** Change in lesion area over time. Data represent mean ± standard error (SE) of triplicate assays. **(C)** Changes in super oxide dismutase (SOD) activity over time. Data represent mean ± SE of triplicate assays. The line charts were generated based on IBM SPSS Statistics 20. The “*” represents the significant differences in the SOD activity.

Water-soaked lesions of the same diameter (0.5 cm) as the plug used for inoculation were observed at 12 hpi (Figure 1A). Over time, the lesions first became rotten and brown (36 hpi) and then turned necrotic (48–72 hpi). The area of lesions expanded with time after inoculation (Figure 1B). Changes in SOD activity were also observed in inoculated leaves (Figure 1C). Significant differences were detected in SOD activity between the control_6 h and inoculated_6 h samples. Superoxide dismutase activity declined before 8 hpi but then increased over time. The sampling time points for metabolomics and transcriptomics were determined based on both the disease symptoms and SOD activity. Given that transcriptional changes usually precede the associated physiological and phenotypic changes, here we considered 6 hpi as the early stage of infection. As the regular ellipse-shaped lesions formed, the 24- and 48-hpi time points were considered as the middle and late stages stage of infection, respectively, for sampling.

### Global Metabolomic Changes

To determine the metabolic changes induced by *B. elliptica* infection, we performed non-targeted metabolome analysis using control_6 h, control_24 h, control_48 h, inoculated_6 h, inoculated_24 h, and inoculated_48 h samples. A total of 524 metabolites common to all samples were identified based on their chromatographic and mass spectrometric parameters (Supplementary Table 2). Orthogonal partial least squares discriminant analysis and principal components analysis (PCA) uncovered differences in the metabolites of all samples (Supplementary Figures 1, 2). High correlation was observed among the QC samples (Supplementary Figure 3). Pearson correlation coefficients were consistently high (*r* > 0.819) across all three biological replicates (Supplementary Figure 4). Heat map, based on the hierarchical clustering analysis of metabolite levels, revealed significant differences in metabolites levels between inoculated and control treatments, and these differences became more pronounced over time (Supplementary Figure 5).

The DAMs were similarly identified by comparing the different temporal stages of pathogen infection, with cutoff values of log_2_ FC ≥ 1 and VIP score ≥ 1. Venn diagram shows the overlap of DAMs among the three stages of infection (Supplementary Figure 6; Supplementary Table 3). A total of 115 DAMs were detected, of which 8, 12, and 62 DAMs were expressed only at the 6-, 24- hpi, and 48-hpi time points. Only eight DAMs, including benzyl salicylate, eriodictyol, tryptamine, esculetin, butin, caffeic acid, dihydrokaempferol, and N'-feruloyl putrescine, were common to all three infection stages (Supplementary Figure 7). Among these eight DAMs, five were involved in phenolic acid metabolism and are known as important secondary metabolites for pathogen resistance (Supplementary Figure 7). Moreover, the accumulation of 15 metabolites was up-regulated and that of 6 metabolites was down-regulated in inoculated_6 h compared with control_6 h (Supplementary Figure 8). Similarly, 39 and 4 metabolites were up-regulated and down-regulated, respectively, in inoculated_24 h compared with control_24 h, and 85 and 5 metabolites were up-regulated and down-regulated, respectively, in inoculated_48 h compared with control_48 h (Supplementary Figure 8). Furthermore, according to KEGG enrichment analysis, the DAMs were enriched in phenylpropanoid biosynthesis, flavonoid biosynthesis, metabolic pathway, and biosynthesis of secondary metabolites at all three infection stages. Phenylpropanoid biosynthesis was significantly enriched at 6 hpi (Supplementary Figure 9A); tryptophan and indole alkaloid biosynthesis were significantly enriched at 24 hpi (Supplementary Figure 9B); and isoflavonoid biosynthesis, fructose and mannose metabolism, and galactose metabolism were significantly enriched at 48 hpi (Supplementary Figure 9C).

### Global Transcriptomic Changes

Samples were used for non-targeted metabolome analysis were subjected to RNA-seq to profile the genome-wide changes in gene expression upon the inoculation of leaves with *B. elliptica*. After removing low-quality reads, 435.16 Gb clean reads were obtained, with an average GC content of 49.87% (Supplementary Table 4). A total of 430,835 transcripts were obtained, averaging 701 bp in length, with N50 and N90 values of 1,213 and 266 bp, respectively (Supplementary Table 5). Using KEGG, NR, Swiss-Prot, GO, and Trembl databases, a total of 283,213 unigenes, with an average length of 534 bp, were functionally annotated (Supplementary Tables 5, 6). In the NCBI NR database, *Asparagus officinalis* (8.94%), *Elaeis guineensis* (8.72%), *Phoenix dactylifera* (7.39%), *Vitis vinifera* (3.63%), and *Cajanus cajan* (3.45%) gave the top BLASTx hits (Supplementary Figure 10). Hierarchal clustering analysis was performed to examine the significant changes in unigene expression. The results showed a stage-specific transcriptome profile after inoculation with *B. elliptica* (Supplementary Figure 11). To validate the RNA-seq data, the expression profiles of 10 defense-related DEGs were evaluated in inoculated and control leaves at 6, 12, 24, 36, and 48 hpi by qRT-PCR (Supplementary Figure 12). All RNA-seq data can be downloaded from NCBI (BioProjects: PRJNA742853).

### Time-Course RNA-Seq Analysis

**Figure 2:**
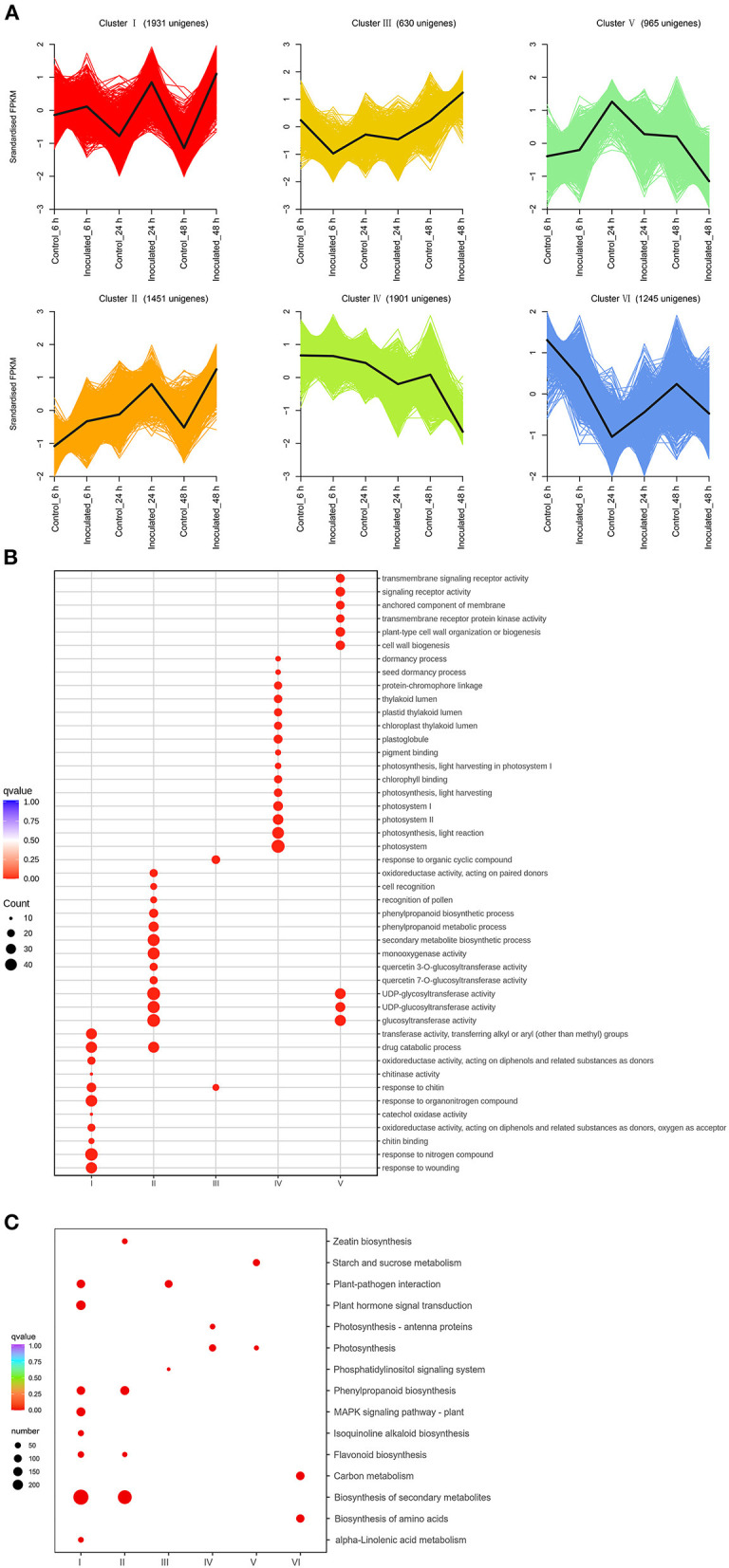
Transcript abundance of all unigenes identified in “Sorbonne”. **(A)** K-means clustering analysis of differentially expressed genes (DEGs), according to their expression profiles. **(B)** Comparison of the gene ontology (GO) enrichment of all unigene clusters. The sizes of dots are proportional to the number of genes per GO term. Only the GO terms with more than 10 DEGs were identified; accordingly, the GO enrichment of cluster VI was eliminated. **(C)** Enrichment of KEGG annotations of DEGs in *Lilium “*Sorbonne” with *B. elliptica* infection. Only the representative and significant pathways among the five clusters are shown in the figure. The size of the circle indicates the quantity, and its color corresponds to the q-value.

The DEGs were identified by comparing the RNA-seq data of inoculated leaf samples (inoculated_6 h, inoculated_24 h, and inoculated_48 h) with those of control leaf samples (control_6 h, control_24 h, and control_48 h) using cutoff values of log_2_(FC) ≥ 1 and Padj ≤ 0.05. All DEGs identified at the three temporal stages were analyzed using the K-means clustering algorithm. The DEGs could be grouped into six clusters and classified into four types (Figure 2A): up-regulated (clusters I, II); down-regulated (clusters IV, V); first up-regulated, then down-regulated (clusters VI); and first down-regulated, then up-regulated (cluster III) (Figure 2A). Gene ontology enrichment analysis showed that one cytomembrane-related term, two chloroplast-related terms, two cytoderm-related terms, three signal transduction receptor-related terms, and six photosynthesis-related terms were enriched among the down-regulated genes (Figure 2B). This suggests that certain biological processes, such as photosynthesis, chlorophyll synthesis, cell wall biogenesis, anchored component of membrane, and signal transduction, might be inhibited by the destruction of leaf tissue by the necrotrophic pathogen. The up-regulated gene clusters were found to be related to chitinase, oxidoreductase activity, cell recognition, secondary metabolites, and the phenylpropanoid biosynthetic process, pointing to their potential positive role in the production of resistant metabolites and proteins in *B. elliptica*-inoculated leaves. The results of KEGG enrichment analysis were consistent with those of GO enrichment analysis: Clusters I and II were mainly enriched in the phenylpropanoid biosynthesis, flavonoid biosynthesis, plant hormone signal transduction, and plant–pathogen interaction pathways; clusters IV and V were mainly enriched in the pathways of photosynthesis and starch and sucrose metabolism (Figure 2C). Taken together, the up-regulated clusters resolved by the K-means clustering algorithm potentially play a pivotal part in the resistance to *B. elliptica*, while the down-regulated clusters related to photosynthesis, cell wall biosynthesis, and other metabolic pathways seem to be negatively affected by *B. elliptica*.

A Venn diagram was used to demonstrate the DEGs identified at all three stages. Overall, 5,295 DEGs were detected, of which 125, 434, and 3,089 were uniquely expressed at 6, 24, and 48 hpi, respectively, and 72 DEGs were common to all three stages (Supplementary Figure 13; Supplementary Table 7). Kyoto Encyclopedia of Genes and Genomes enrichment analysis was performed to further characterize these DEGs. At 6 hpi, 401 DEGs (86 up-regulated, 315 down-regulated) were significantly enriched in “biosynthesis of secondary metabolites”, “biosynthesis of amino acid”, and “carbon metabolism” pathways (Supplementary Figures 14, 15A). At 24 hpi, 1,887 DEGs (309 up-regulated, 1,578 down-regulated) were significantly enriched in six pathways: “biosynthesis of secondary metabolites”, “plant hormone signal transduction”, “phenylpropanoid biosynthesis”, “flavonoid biosynthesis”, “isoquinoline alkaloid biosynthesis”, and “MAPK signal pathway” (Supplementary Figures 14, 15B). At 48 hpi, 4,726 DEGs (2,772 up-regulated, 1,954 down-regulated) were significantly enriched in five pathways: “biosynthesis of secondary metabolites”, “plant hormone signal transduction”, “plant–pathogen interaction”, “flavonoid biosynthesis”, and “carbon metabolism” (Supplementary Figures 14, 15C). Taken together, these results indicate that “biosynthesis of secondary metabolites”, “flavonoid biosynthesis”, “phenylpropanoid biosynthesis”, “plant hormone transduction signal”, “plant-pathogen interaction”, and “MAPK signal pathway” were the chief pathways underpinning the responses of “Sorbonne” to *B. elliptica* infection at the transcript level.

### Role of Phenylpropanoid and Flavonoid Biosynthesis Pathways in the *Lilium* Defense Response

**Figure 3:**
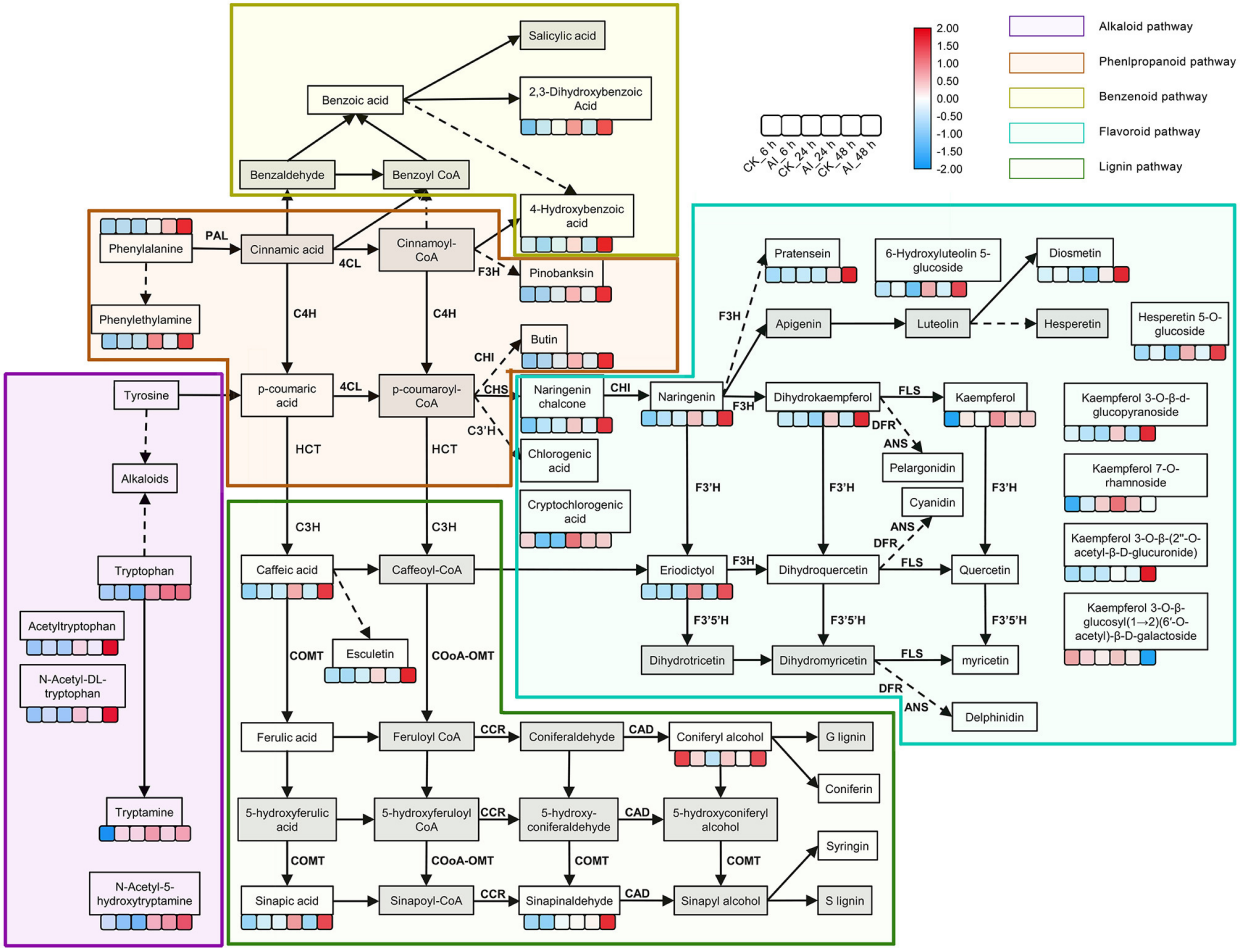
Summary of metabolite changes in *Lilium* “Sorbonne” caused by *B. elliptica* infection. The metabolites not detected are indicated by a gray-filled grid. Dotted lines indicated some metabolites, and enzymes are not shown. Metabolites with statistically significant (*P* ≤ 0.05) changes are shown in the heat map, in which blue and red colors indicate low and high accumulation, respectively. Data on the number of metabolites in the heat map were log-transformed. The six boxes represent control (uninoculated) and inoculated samples at different stages of infection (i.e., different time points post-inoculation): control_6 h, inoculated_6 h, control_24 h, inoculated_24 h, control_48 h, and inoculated_48 h (left to right). Different background colors represent different metabolic pathways: purple, alkaloid pathway; brown, phenylpropanoid pathway; yellow green, benzenoid pathway; blue, flavonoid pathway; green, lignin pathway. Gene names, annotations, and RNA-seq data are summarized in Supplementary Figure 15. PAL, phenylalanine ammonia-lyase; C4H, cinnamic acid 4-hydroxylase; 4CL, *p*-coumaroylCoA ligase; HCT, hydroxycinnamoyl transferase; CSE, caffeoylshikimate esterase; C3′H, coumaroylquinate 3′-monooxygenase; COMT, caffeic acid *O*-methyltransferase; CCoAOMT, caffeoyl coenzyme A 3-*O*-methyltransferase; CCR, cinnamoyl-CoA:NADPH oxidoreductase; CAD, cinnamyl alcohol dehydrogenase; CHS, chalcone synthase; CHI, chalcone isomerase; F3H, naringenin 3-dioxygenase; F3′H, flavonoid 3′-monooxygenase; FLS, flavonol synthase; DFR, dihydroflavonol 4-reductase; ANS, anthocyanidin synthase.

The phenylpropanoid and flavonoid biosynthesis pathways could be integrated into a transcriptional cascade and metabolic network, which together play a critical role in how the *Lilium* hybrid “Sorbonne” responds to *B. elliptica* infection. All the DEGs and DAMs participating in the phenylpropanoid and flavonoid biosynthesis pathways that exhibited up- or down-regulated trends in the different infection stages were selected and mapped to this network (Figure 3; Supplementary Figure 16).

Phenylalanine is the first metabolite in the phenylpropanoid pathway. Although our non-targeted metabolome analysis indicated a significant difference in its accumulation between the inoculated and control treatments at 48 hpi, we identified significant expression of *phenylalanine ammonia-lyase* (*PAL*), a structural gene that catalyzes phenylalanine, clustering at 24 hpi. The expression of *PAL* (DN129152_c1_g2) in the inoculated treatment was 3.5- and 6.5-fold higher at 24 and 48 hpi, respectively, compared with the control (Supplementary Figure 16; Supplementary Table 2), implying that *PAL* participates in the defense response. Moreover, the levels of phenylalanine derivatives, such as phenethylamine and 4-hydroxybenzoic acid, were significantly higher in the inoculated leaves at 24 and 48 hpi compared with the control, indicating that the phenylpropanoid pathway was activated by the infection. Besides, the same expression pattern was exhibited by the structural gene (DN134629_c3_g2) that catalyzes 4-hydroxybenzoic acid. The accumulated level of caffeic acid, a precursor of the lignin pathway, was 1.6-, 3.8-, and 11-fold higher in inoculated leaves at 6, 24, and 48 hpi, respectively, compared with the control (Supplementary Table 2). The level of esculetin, a downstream metabolite of caffeic acid, increased rapidly in leaves after inoculation (Supplementary Table 2). Among the downstream metabolites of caffeic acid, ferulic acid and coniferyl alcohol, which act as precursors of guaiacyl, were down-regulated at 6 hpi but up-regulated at 24 hpi and 48 hpi. Sinapinaldehyde and sinapic acid, precursors of syringyl lignin, were up-regulated in all three stages. The accumulation of syringaresinol-hex and syringaresinol, downstream metabolites of S-lignin, increased significantly in leaves post inoculation (Supplementary Table 2).

The accumulation of naringenin chalcone, naringenin, and dihydrokaempferol, which represent metabolites in the flavonoid biosynthesis pathway, increased significantly at different stages of infection. Additionally, the expression of *chalcone synthase* (*CHS*), a structural gene that promotes the synthesis of the three abovementioned metabolites, was induced at all three stages (Supplementary Figure 16; Supplementary Table 2). Among all the DAMs detected in the flavonoid biosynthesis pathway, the greatest difference in accumulation occurred in eriodictyol, which was undetectable in all the control treatments and was 5- and 37-fold higher at 24 and 48 hpi, respectively, than at 6 hpi in the inoculated treatments (Supplementary Table 2). The accumulation level of kaempferol in the inoculated treatment was 190- and 14-fold higher than that in the control treatment at 6 and 24 hpi, respectively, but its level was similar between the inoculated and control treatments at 48 hpi (Supplementary Table 2). The expression of *UDP-glycosyltransferase* (DN130728_c1_g2) in the inoculated treatment increased significantly at 6 hpi, which could potentially explain the accumulation of kaempferol derivatives at 24 and 48 hpi. The accumulation of four downstream metabolites of kaempferol, namely kaempferol 3-O-β-d-glucopyranoside, kaempferol 3-O-β-glucosyl(1 → 2)(6′-O-acetyl)-β-D-galactoside, kaempferol 7-O-rhamnoside, and kaempferol 3-O-β-(2″-O-acetyl-β-D-glucuronide), was significantly increased in the inoculated treatment at 48 hpi compared with the corresponding control (Figure 3). In addition, the accumulation of the derivatives of luteolin (6-hydroxyluteolin 5-glucoside) and hesperetin (hesperetin 5-O-glucoside) in the inoculated treatment increased significantly at 24 and 48 hpi. The level of diosmetin, a downstream product of luteolin, was five-fold higher in the inoculated treatment than in the control treatment.

### Potential Transcriptional Regulatory Mechanisms

**Figure 4:**
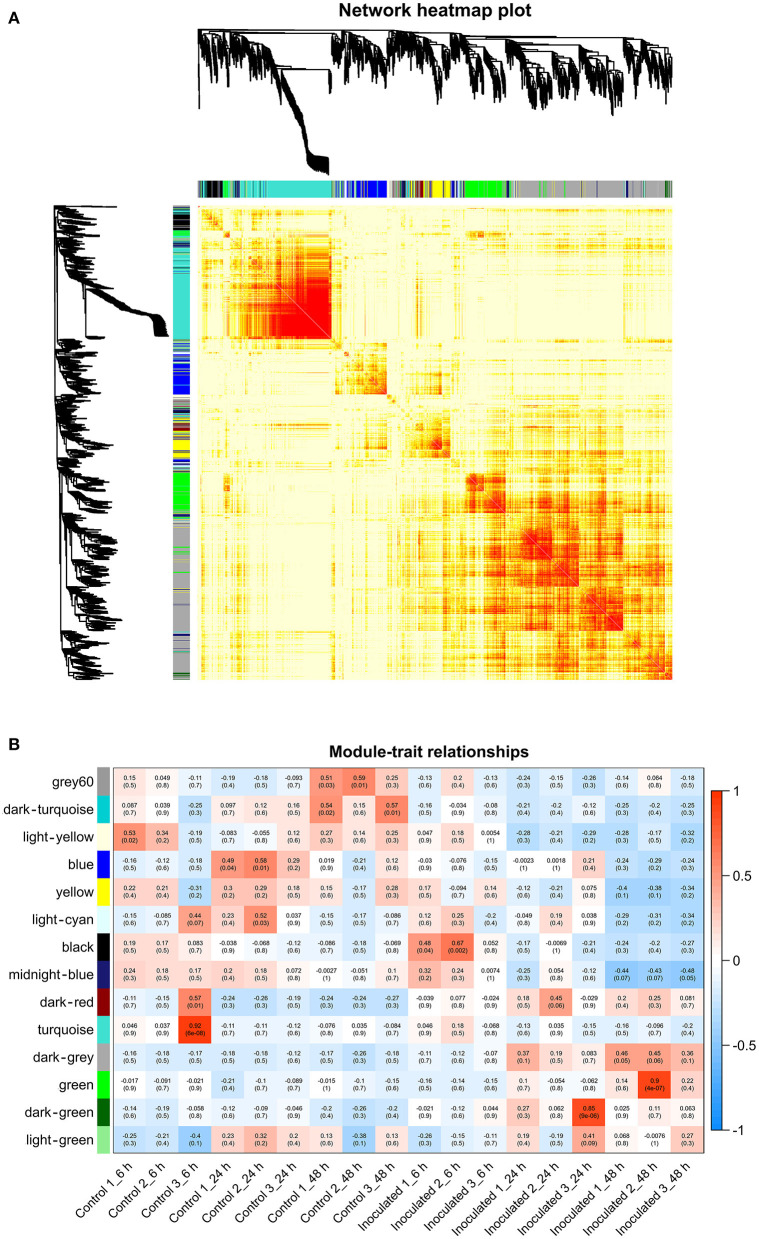
Results of weighted gene coexpression network analysis (WGCNA) of “Sorbonne” transcripts. **(A)** Dendrogram of genes, based on co-expression network analysis. The gene dendrogram was obtained by hierarchical clustering analysis, with the module color indicated by the color of the row underneath. A total of 14 distinct modules were identified. **(B)** Association between modules and plant defense traits. The color of each module is the same as that in **(A)**. Each row in the table corresponds to a module, and each column corresponds to a sample. Values in each cell indicate the number of corresponding correlations and their *P*-values.

**Figure 5:**
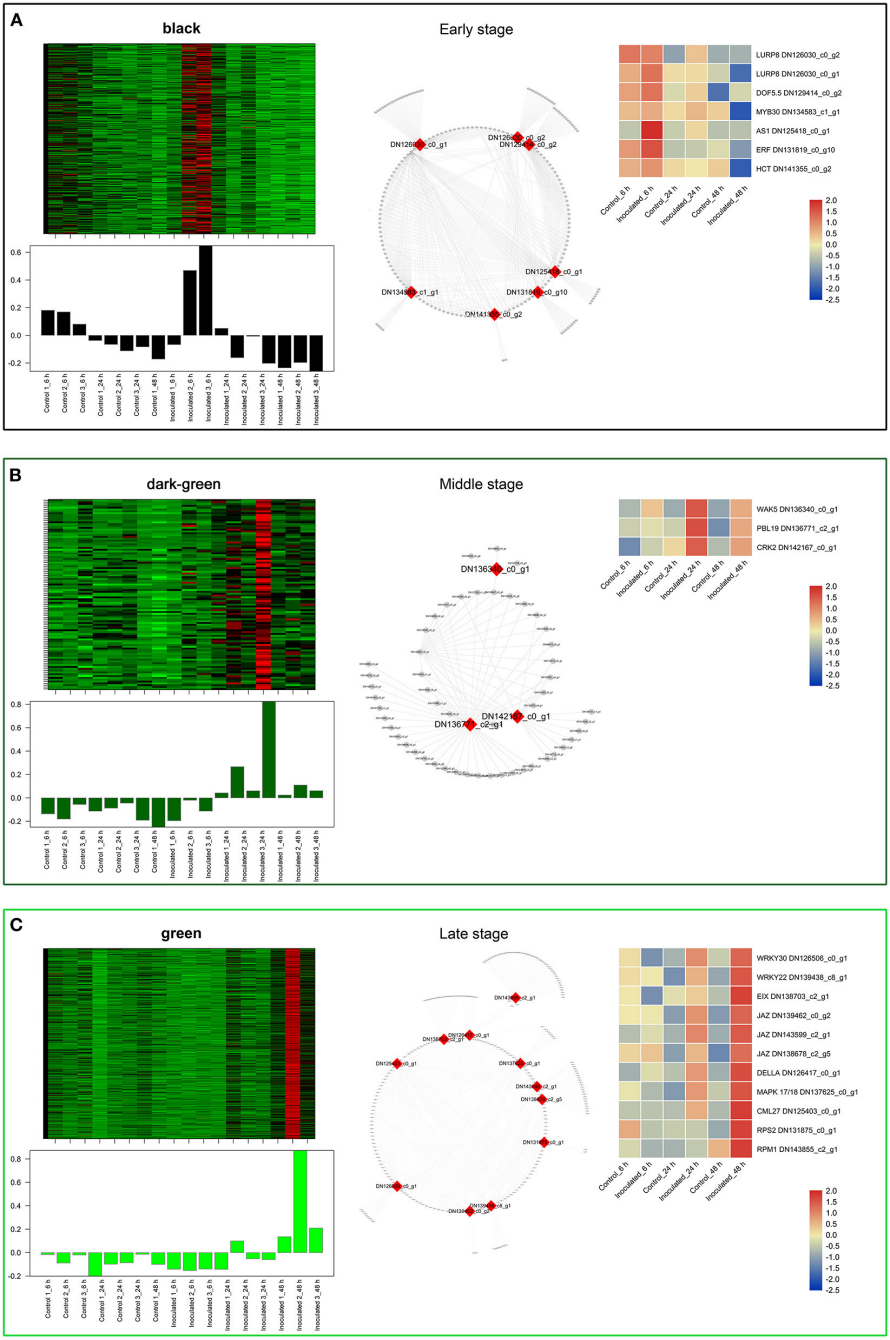
“Sorbonne” DEGs identified at different stages of *B. elliptica* infection. **(A–C)** Co-expression network of the black module **(A)**, dark-green module **(B)**, and green module **(C)**. Heat maps and bar graphs show the co-expressed genes in each module (left). Red rectangles on the heat map denote high expression levels; green rectangles denote low expression levels. The network of top hub genes is indicated by red squares in the network (middle). Heat maps (right) show the expression patterns of hub genes.

**Figure 6:**
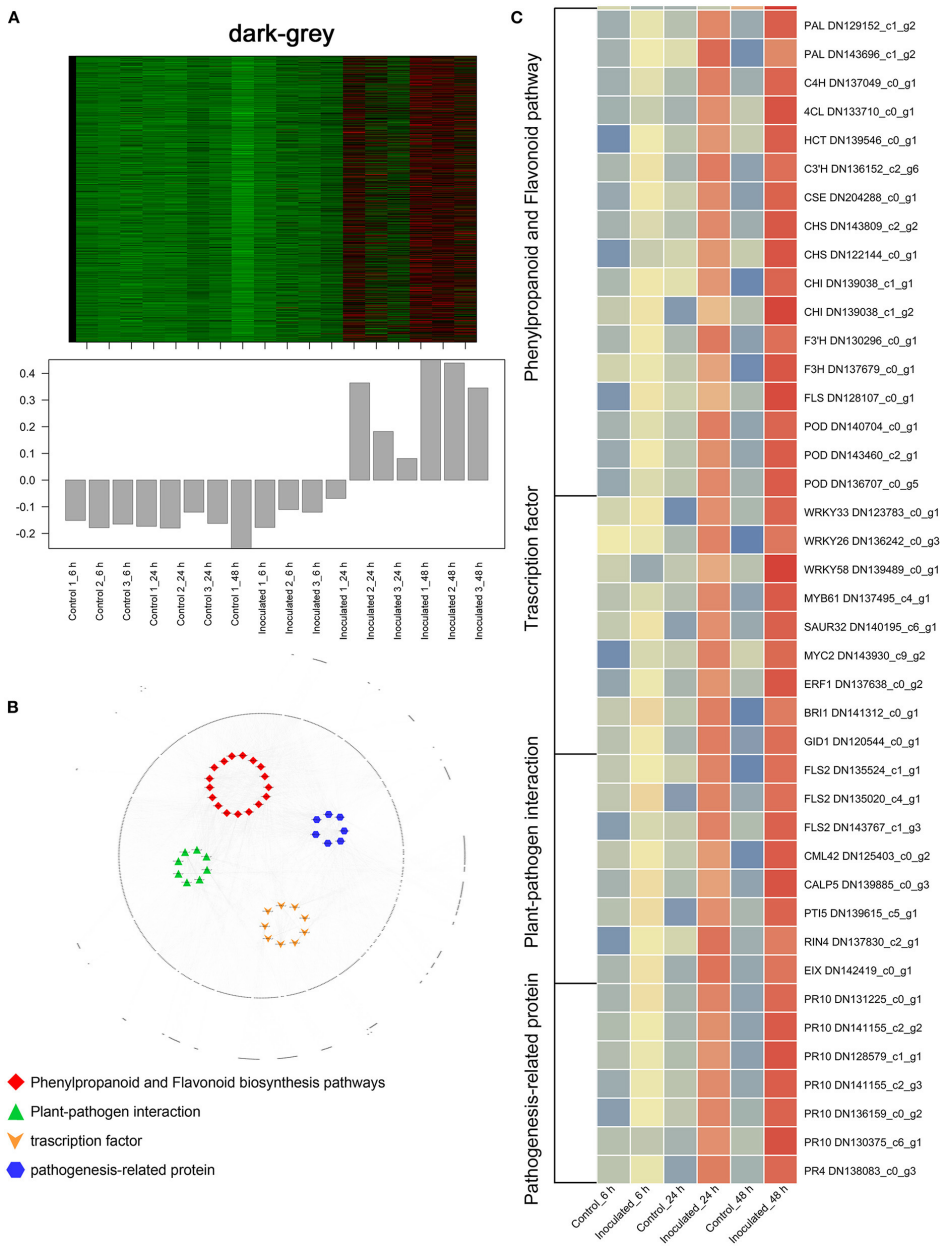
“Sorbonne” DEGs common to all three stages of *B. elliptica* infection. **(A)** Heat maps and bar graphs show the co-expressed genes in each module. Red rectangles denote high expression levels; green rectangles denote low expression levels. **(B)** Network of hub genes is indicated by larger font size in the network. Small dots around the hub genes represent other co-expressed genes, and their relationships are connected by lines. Red squares represent the phenylpropanoid and flavonoid pathways; green triangles represent plant–pathogen interactions; orange arrows represent transcription factors (TFs); blue hexagons represent pathogenesis-related (PR) proteins. **(C)** Heat maps showing the expression patterns of hub genes.

To investigate the gene regulatory network of *Lilium* Sorbonne after its inoculation with *B. elliptica*, we performed WGCNA of all DEGs identified from the RNA-seq analysis of the 18 cDNA libraries. After preprocessing the RNA-seq data, 14 gene co-expression modules, each comprising 80–2,659 genes, were discovered (Figure 4A). Module-trait and sample relationship analyses showed the eigengenes of these modules were correlated with the different infection stages. Unlike most modules in which the trend of genes was that of almost no difference between the inoculated and control treatments, a few modules did differ significantly during the defense response (Figure 4B). Next, we identified key genes (i.e., hub genes) that played crucial roles during the infection, based on the WGCNA for these notable modules. The levels of gene expression in the black, dark-green, and green modules showed peak up-regulation at 6, 24, and 48 hpi, respectively, which correspond to the early, middle, and late stages of infection, respectively (Figure 5). Gene expression in the dark-gray module was higher in the inoculated treatment than in the control treatment at all-time points, indicating that genes in this module play a prominent role in the defense response against *B. elliptica* (Figure 6).

In the black module (Figure 5A), seven hub genes related to plant defense were identified, such as two *LURP* homologs (DN126030_c0_g1, DN126030_c0_g2), one *MYB30* homolog (DN134583_c1_g1), and one *AS1* homolog (DN125418_c0_g1). Among these, *DOF5.5* (DN129414_c0_g2) showed a regulatory relationship with the three defense response-related hub genes. In addition, one *WRKY70* gene (DN139965_C0_G2) showed significant difference in expression between inoculated and control treatments at 6 hpi. Additionally, an *ERF* homolog (DN131819_c0_g10) and a *HCT* homolog (DN141355_c0_g2) were up-regulated at the early stage of infection. In the dark-green module (Figure 5B), we identified three hub genes related to plant–pathogen interactions: a *PBL19* homolog (DN136771_c2_g1), a *CRK2* homolog (DN142167_c0_g1), and a *WAK5* homolog (DN136340_c0_g1). Notably, *PBL19* showed a strong regulatory relationship with other two hub genes, *CRK2* and *WAK5*. A *CKX9* homolog (DN129547_c0_g1), a *I2*′*H* homolog (DN140336_c1_g1), and a *CAD* homolog (DN127300_c1_g1) were significantly up-regulated in the dark-green module. At the late stage of the infection (Figure 5C), 11 hub genes were identified, including a *WRKY22* homolog (DN139438_c8_g1), a *WRKY30* homolog (DN126506_c0_g1), three *JAZ* homologs (DN139462_c0_g2, DN143599_c2_g1, DN138678_c2_g5,), a *DELLA* homolog (DN126417_c0_g11), a *MAPK17/18* homolog (DN137625_c0_g), and a *CML27* homolog (DN125403_c0_g1). Furthermore, both an *RPS2* homolog (DN131875_c0_g1) and an *RPM1* homolog (DN143855_c2_g1) displayed strong regulatory relationships with other genes in the green module. Genes in the dark-gray module (2,659, i.e., more than half of all identified DEGs) were up-regulated at all three infection stages (Figure 6A). Analysis of DEGs revealed 41 hub genes, which included a *MYB61* homolog (DN137495_c4_g1), a *WRKY33* homolog (DN123783_c0_g1), *MYC2* homolog (DN143930_c9_g2), and a *BRI1* homolog (DN141312_c0_g1), among others (Figures 6B,C). A number of hub genes were structural genes involved in the phenylpropanoid and flavonoid biosynthesis pathways (e.g., two *PAL* homologs [DN129152_c1_g2, DN143696_c1_g2], an *HCT* homolog [DN139546_c0_g1], two *CHS* homologs [DN143809_c2_g2, DN122144_c0_g1], an *F3*′*H* homolog [DN130296_c0_g1], and a *FLS* homolog [DN128107_c0_g1], and plant–pathogen interactions (e.g., three *FLS2* homologs [DN135524_c1_g1, DN135020_c4_g1, DN143767_c1_g3] and an *RIN4* homolog [DN137830_c2_g1], in addition to several *PR* genes. Lastly, a gene called *Pti5* (DN139615_c5_g1), which encoded an AP2-EREBP family TF, also exerted a great influence on plant–pathogen interactions.

## Discussion

### The Central Role of Phenylpropanoid and Flavonoid Biosynthesis Pathways in the Defense Response of *Lilium*

Phenolic compounds, such as metabolites of the phenylpropanoid and flavonoid pathways, act as fungitoxic and antimicrobial defense compounds in host–pathogen interactions (Martinez et al., 2017; Xu et al., 2018, 2019). Caffeic acid is a key metabolite in the phenylpropanoid pathway. In our study, significantly more caffeic acid accumulated over time in the inoculated treatment than in the control treatment. Consistent with this result, the structural genes involved in the synthesis of caffeic acid (*HCT* and *CSE*) were significantly up-regulated in the inoculated treatment (Figure 3; Supplementary Figures 12, 16). In apple, caffeic acid has been shown to effectively promote lignin accumulation and inhibit gray mold infection (Zhang et al., 2020). Although the accumulation of lignin was not detected in our study, we detected significant accumulation of the precursors precursor of G-lignin (coniferyl alcohol) and S-lignin (sinapic acid and sinapinaldehyde) (Figure 3). The structural genes for the synthesis of lignin, *COMT, CCR*, and *CAD*, were also significantly up-regulated in the inoculated treatment (Supplementary Figure 16). Additionally, two transcripts, DN141508_c1_g3 and DN141508_c1_g4, encoding the CCoAOMT enzyme, were significantly up-regulated at all stages of infection (Supplementary Table 7). CCoAOMT is reportedly a lignin synthase that can enhance the resistance of *Lilium* to *B. cinerea* infection (Fu et al., 2020). Our results showed that caffeic acid may enhance the disease resistance of *Lilium* by modulating the lignin biosynthetic pathway.

In our network, the flavonoid pathway begins with naringenin chalcone to produce naringenin, which is then converted into apigenin, dihydrokaempferol, and eriodictyol (Figure 3). Subsequently, apigenin and dihydrokaempferol are converted into luteolin and kaempferol, respectively (Figure 3). The entire pathway was specifically activated in *Lilium* after infection with *B. elliptica*, as evident from the up-regulation of several structural genes, including *CHS, CHI, F3*′*H*, and *FLS* (Supplementary Figures 12, 16). Eriodictyol was recently identified as a novel antibacterial compound; however, its role in plant–pathogen interactions has not yet been determined (Ho et al., 2018). In the current study, eriodictyol accumulation showed significant differences between the inoculated and control treatments at all stages of infection. Interestingly, evidence shows that eriodictyol synthase *F3*′*H* (DN130296_c0_g1) enhances host plant resistance against invading pathogens (Mizuno et al., 2014; Hutabarat et al., 2016). Further analysis of eriodictyol may thus open new opportunities for the cultivation of resistant *Lilium*. Despite no significant difference in the accumulation of kaempferol at the late stage of infection, the expression of its biosynthetic gene *FLS* (DN128107_c0_g1) and conjugated compounds, which can be stored in vacuoles for long periods, increased with time post-inoculation. FLS and F3′H are responsible for the synthesis of kaempferol and quercetin, and were recently shown to be critical for disease resistance in plants (Zhang et al., 2016). Together, these findings suggest the possibility that kaempferol and eriodictyol are the main metabolites of the flavonoid pathway that shape the response of *Lilium* to *B. elliptica* infection, and their levels are affected by a series of enzymes such as CHS, F3H, and FLS.

Analysis of the time-course RNA-seq data and WGCNA results also support the central role of phenylpropanoid and flavonoid biosynthesis pathways in the defense response of *Lilium*. At the early stage of infection, the *MYB30* gene was significantly up-regulated in inoculated leaves (Figure 5A). The MYB TFs have been shown to confer resistance to rice plants by regulating the phenylpropanoid pathway (He et al., 2020). Consistently, the accumulation of phenylpropanoid pathway-related metabolites and the expression of *MYB30* significantly increased at the early stage of pathogen infection. At the middle stage, *CAD* and *I2*′*H*, which represent structural genes in the lignin and flavonoid pathways, respectively, were significantly up-regulated in the dark-green module (Figure 5B). At all three stages, *MYB61* was highly up-regulated, which affected the expression of structural genes involved in the phenylpropanoid and flavonoid pathways, such as *PAL, CSE, CHS, CHI*, and *POD* (Figure 6). This finding is consistent with the significant accumulation of caffeic acid, dihydrokaempferol, and kaempferol in the inoculated treatments at the three stage of infection.

### Role of Hormone Signaling Pathways in *B. elliptica*–“Sorbonne” Interaction

Analysis of the transcriptome time series and WGCNA results let us further elucidate important signaling pathways underlying the defense responses at transcriptional level. In *B. elliptica*-inoculated leaves with no lesions, the defense regulator gene *DOF5.5* was significantly up-regulated, indicating its involvement in the plant response to infection, similar to that of *MYB30* and *AS1* (Figure 5A). The DOF TFs function as upstream regulators of the salicylic acid (SA) signaling pathway, and enhance the resistance to pathogens in plants (Kang et al., 2003, 2016; Yu et al., 2019). In *Arabidopsis*, changes in *MYB30* expression levels modulated the SA content and SA-associated gene expression levels (Raffaele et al., 2006). Interestingly, the WGCNA results showed \ a regulatory relationship between *DOF5.5* and *AS1*. The *AS1* gene acts a positive regulator of SA-independent extracellular defenses but a negative regulator of the defense response by selectively binding to the promoters of genes controlled by the immune activator, jasmonic acid (JA) (Nurmberg et al., 2007). Additionally, *WRKY70* differed significantly between the inoculated and control treatments at the early stage of infection. The *AtWRKY70* is an important node of convergence for the SA- and JA-mediated defense signaling pathways (Li et al., 2017).

With the expansion of necrotic lesions on *B. elliptica*-inoculated “Sorbonne” leaves (middle stage of infection), genes including *PBL19, CRK2*, and *WAK5*, which are all members of the Interleukin-1 Receptor-Associated Kinase (IRAK) gene family, were instrumental in the defense against *B. elliptica* (Figure 5B). The IRAK family proteins are conserved upstream signaling molecules that can regulate various stress adaptation programs in plants (Srideepthi et al., 2020). Our results demonstrated a strong correlation between the expression of *PBL19* and *CKX9*. OsPBL1 exhibits 67% amino acid sequence identity to a positive regulator of ETI, and the expression of *OsPBL1* in transgenic *Arabidopsis* increased after an exogenous treatment of cytokinin and SA (Lee and Kim, 2015). *CKX5*, the only known gene involved in cytokinin catabolism, was recently proven to respond to *B. cinerea* infection in various ways that are differently modulated by JA and ethylene biosynthesis pathways in *Arabidopsis* (Li et al., 2021).

At the late stage of *B. elliptica* infection (green module), necrotic lesions on leaves expanded, and *JAZ, WRKY30*, and *WRKY22* genes played central roles in plant defense (Figure 5C). The *JAZ* genes encode transcriptional repressors of JA-responsive genes and major components of the JA receptor complex (Thatcher et al., 2016). *WRKY30* and *WRKY22* are known to enhance disease resistance in plants via the SA and JA defense systems (Peng et al., 2012; Han et al., 2013; Jiang et al., 2015; Kloth et al., 2016).

Furthermore, several differentially expressed TF-encoding genes were identified in the dark-gray module, indicating that these TFs may be significantly involved in the defense response at all three stages (Figure 6). The results revealed a major TF involved in SA and JA signaling, *WRKY33* (Figure 5C). *WRKY33* showed a strong regulatory relationship with *MYC2* and *NPR1*, and was highly expressed in the inoculated leaves. *AtWRKY33* plays a major role in the crosstalk between JA and SA signaling pathways and metabolic responses in response to *B. cinerea* infection (Birkenbihl et al., 2012; Liu et al., 2017). *MYC2* positively regulates JA-mediated flavonoid biosynthesis, while *NPR1* modulates SA and JA antagonism in plants (Dombrecht et al., 2007; Knoth et al., 2009). In addition, the *FLS2, BAK1, BRI1*, and *MYB61* genes were significantly up-regulated across all infection stages in our results. *FLS2* is a phylogenetically related cell surface pattern recognition receptor and a coreceptor for *BAK1*. *BAK1* is a coreceptor for the brassinolide (BR) receptor BRI1 (Tian et al., 2014), which is responsible for initiating the events of BR signal transduction. While the BR signaling pathway contributes to the growth–defense tradeoff by suppressing the expression of defensin and glucosinolate biosynthesis genes (Liu et al., 2018; Liao et al., 2020). *MYB61* was also positively regulated by *BRI1* in our results, but whether *MYB61* is involved in BR signaling remains unknown.

Taken together, our analysis indicates that SA and JA signaling pathways play pivotal roles in *B. elliptica–* Sorbonne” interaction. This supports the proposal that a large number of transcripts related to *B. elliptica* resistance in *L. regale* were involved in the JA and phenylpropanoid pathways (Cui et al., 2018a). Moreover, as reported in the interactions between other hosts and *Botrytis* spp. (e.g., *Arabidopsis*–*B. cinerea* and *Arabidopsis*–*Alternaria brassicicola*), the SA and JA signaling pathways play a crucial role in the response of “Sorbonne” to fungal infection (Zheng et al., 2006; Ederli et al., 2015, 2021; Liao et al., 2020). Besides, the BR-related genes were significantly up-regulated during across all infection stages in our results. The crosstalk between BR and JA signaling affects the growth-defense tradeoff in *Arabidopsis*–*B. cinerea* interaction (Liu et al., 2018; Liao et al., 2020), while exogenous BR before the inoculation of *B. cinerea* enhances the defense response in rose petals (Liu et al., 2018). The role of BR pathway in *Lilium* defense response to the gray mold remains to be further elucidated.

### Other Signal Transduction Pathways That Contribute to the *Lilium* Defense Response Against *B. elliptica*

In addition to phenolics and hormone signaling pathways, our results showed that other signal transduction pathways play a prominent role in the defense response. At the early stage of stage, *LURP* was significantly up-regulated in the plant response to infection, and regulated the transcript levels of *POD* and *HSP70* (Figure 5A). *POD* and *HSP70* are important for plant resistance (Baig, 2018). *AtLURP1* shows an unusually pronounced transcriptional up-regulation in response to infection (Knoth and Eulgem, 2008). Together, these data suggest that *LURP* plays a pivotal role in the response of “Sorbonne” to *B. elliptica* infection at the early stage.

At the middle stage, the *PBL19, CRK2*, and *WAK5* genes were instrumental in the defense response of “Sorbonne” against *B. elliptica* (Figure 5B). The *pbl13-2* knockout mutant shows higher level of reactive oxygen species (ROS) and greater flagellin-induced activation of mitogen-activated protein kinases (MAPKs) than the wild type (Lin et al., 2015). Moreover, overexpression of *CRKs* enhances PTI in transgenic *Arabidopsis* (Yeh et al., 2015). Wall-associated kinases (WAKs) localize to the cell wall and participate in pathogen recognition and signal transduction in plants (Kurt et al., 2020). Collectively, the transduction of various signals contributed to the defense response of *Lilium* against *B. elliptica* at the middle stage of infection.

At the late stage of infection, *CML27, DELLA*, and *JAZ* played central roles in plant defense. *CML27* encodes a Ca^2+^-binding protein involved in the Ca^2+^ signaling pathway. Ca^2+^ regulates diverse cellular processes and functions as a secondary messenger, enabling plants to sense and quickly respond to extracellular stimuli (Edel et al., 2017). *AtRPS2* and *AtRPM1* activate Ca^2+^-dependent protein kinases (CPKs) for mediating bifurcate immune responses (Gao et al., 2013). In our results, *RPS2* and *RPM1* were significantly up-regulated among the hub genes, indicating the important role of the Ca^2+^ signaling pathway in the defense response of “Sorbonne”.

In all three stages (Figure 6), *MYB61* was highly expressed, implying that it is central to the defense response. *B. elliptica* infects *Lilium* via the stomata, and *MYB61* regulates stomatal aperture (Hsieh et al., 2001; Gao et al., 2018; Romero-Romero et al., 2018), which may explain its role in the defense response. In the current study, the expression levels of *PR10* and *PR4* were strongly correlated with that of *Pti5* (Figure 6). Overexpression of the tomato TF genes, *Pti4/5/6*, in *Arabidopsis* showed that Pti4/5/6 activate the expression of a wide array of *PR* genes *in vivo*, resulting in enhanced defense against certain fungal pathogens (Gu et al., 2002; Gonzalez-Lamothe et al., 2008). The expression levels of *RIN4, RPM1, RPS2, NPR1*, and *TGA* were significantly up-regulated at all infection stages. In *Arabidopsis*, the phosphorylation and cleavage of RIN4 activates *RPM1* and *RPS2*, which encode R proteins involved in ETI (Li et al., 2019; Ray et al., 2019; Yang et al., 2021; Zhao et al., 2021). NPR1-mediated DNA binding of *TGA2* is critical for the activation of defense related genes. Both *NPR1* and *TGA1* act as master redox-sensitive transcriptional regulators of *PR* genes in plants (Fu and Dong, 2013). Altogether, our results suggest the transduction of diverse signals jointly shape the defense response of *Lilium* hybrid “Sorbonne” to *B. elliptica*.

## Conclusion

In this study, we used a non-targeted metabolomic analysis complemented by NGS to understand the defense response of *Lilium* hybrid “Sorbonne” to *B. elliptica* infection. Multivariate data analysis demonstrated that the phenylpropanoid and flavonoid pathways play a central role in the plant defense response. Network analysis revealed high interconnectivity among factors involved in the induced defense response. Furthermore, we performed WGCNA to investigate the DEGs, and identified a number of hub genes at different stages of infection, indicating that JA, SA, BR, and Ca^2+^ also play important roles in the defense response. Thus, our study provides a comprehensive understanding of the defense response of “Sorbonne” to *B. elliptica* infection. Further investigation is needed to elucidate the regulatory mechanisms underlying the defense response of *Lilium*.

## Data Availability Statement

The original contributions presented in the study are publicly available. This data can be found here: National Center for Biotechnology Information (NCBI) BioProject database under accession number PRJNA742853 (https://www.ncbi.nlm.nih.gov/sra/PRJNA742853).

## Author Contributions

DL and NC: conceptualization. ML and NC: methodology. NC, JX, and RZ: formal analysis and investigation. JX, NC, and ZS: writing—original draft preparation. DL, YC, and SS: writing—review and editing. YC: funding acquisition. ML: resources. DL and SS: supervision. All authors contributed to the article and approved the submitted version.

## Funding

This work was supported by the National Key Research and Development Program of China (2018YFD1000407).

## Conflict of Interest

The authors declare that the research was conducted in the absence of any commercial or financial relationships that could be construed as a potential conflict of interest.

## Publisher's Note

All claims expressed in this article are solely those of the authors and do not necessarily represent those of their affiliated organizations, or those of the publisher, the editors and the reviewers. Any product that may be evaluated in this article, or claim that may be made by its manufacturer, is not guaranteed or endorsed by the publisher.
