# Supplementary material for: Metabolic and Transcriptomic Profiling of Lilium Leaves Infected With Botrytis elliptica Reveals Different Stages of Plant Defense Mechanisms
Source: Front Plant Sci. 2021 Sep 22;12:730620. doi: 10.3389/fpls.2021.730620 (PMC8493297; doi:10.3389/fpls.2021.730620)
Supplement: Supplementary file 2 [file Data_Sheet_1.PDF]

Supplementary Material

Supplementary Figures

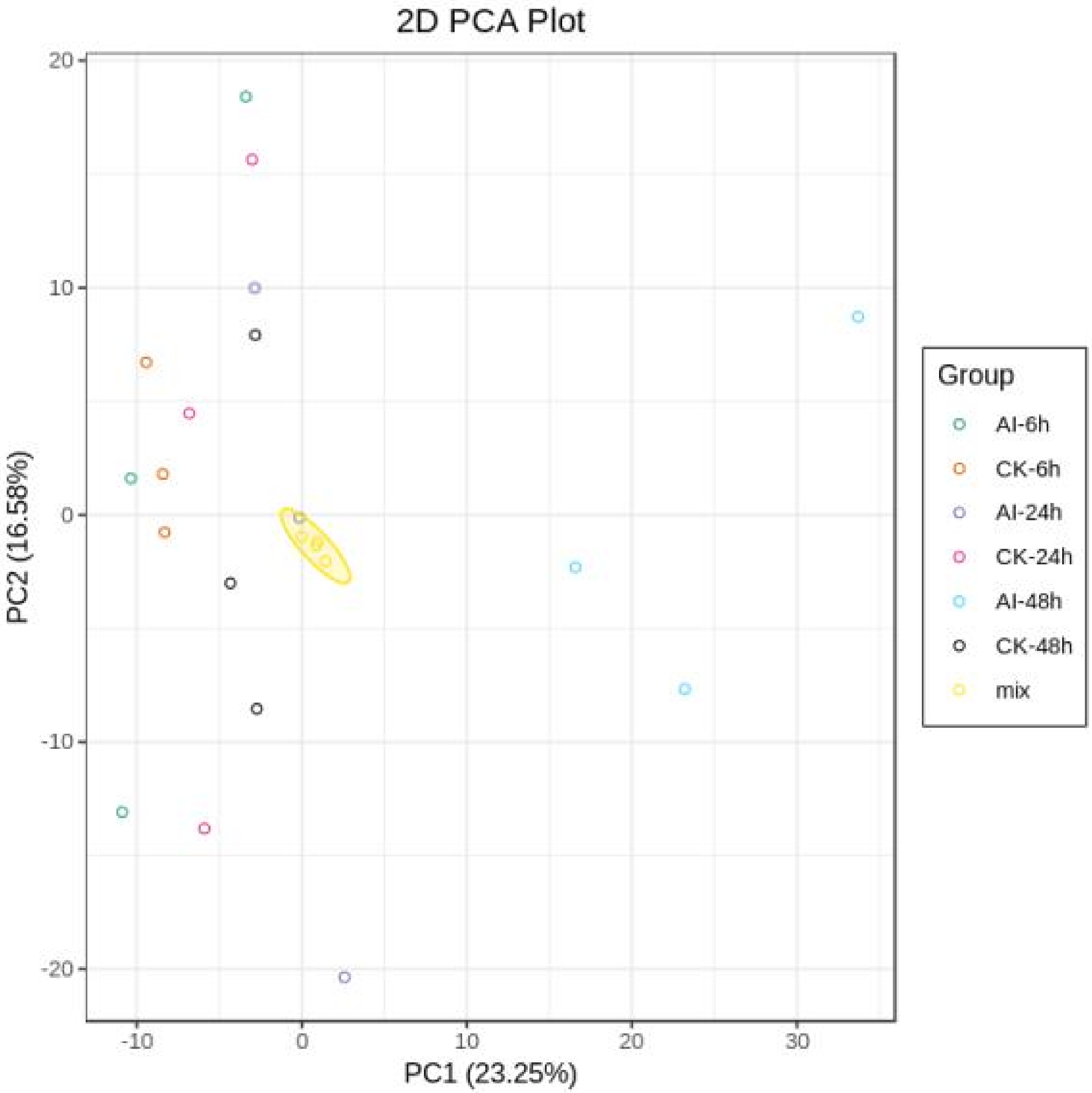

Supplementary Figure 1**.** PCA plots for all samples.

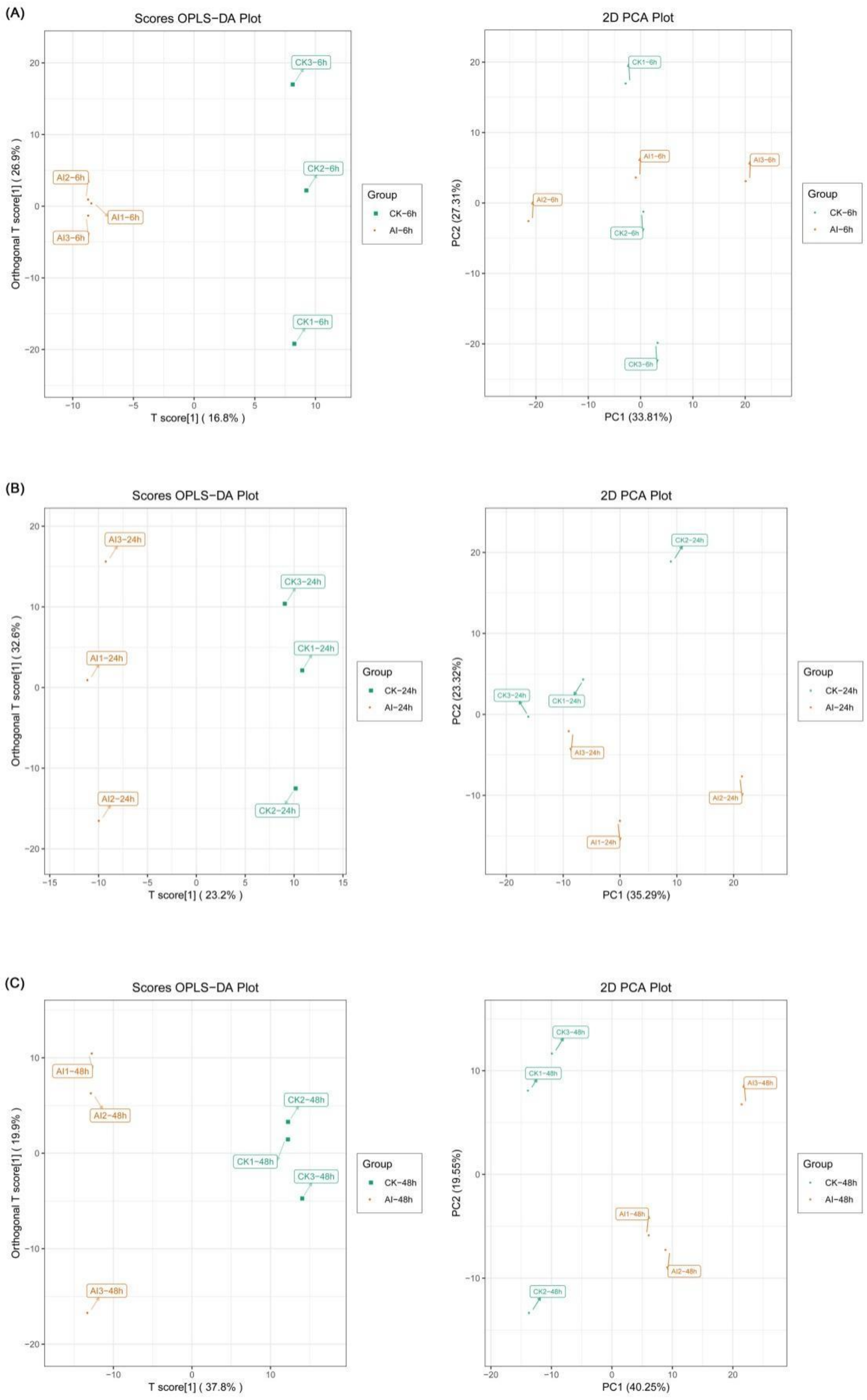

Supplementary Figure 2. PCA plots and OPLS plots at 6 hpi, 24 hpi, and 48 hpi.

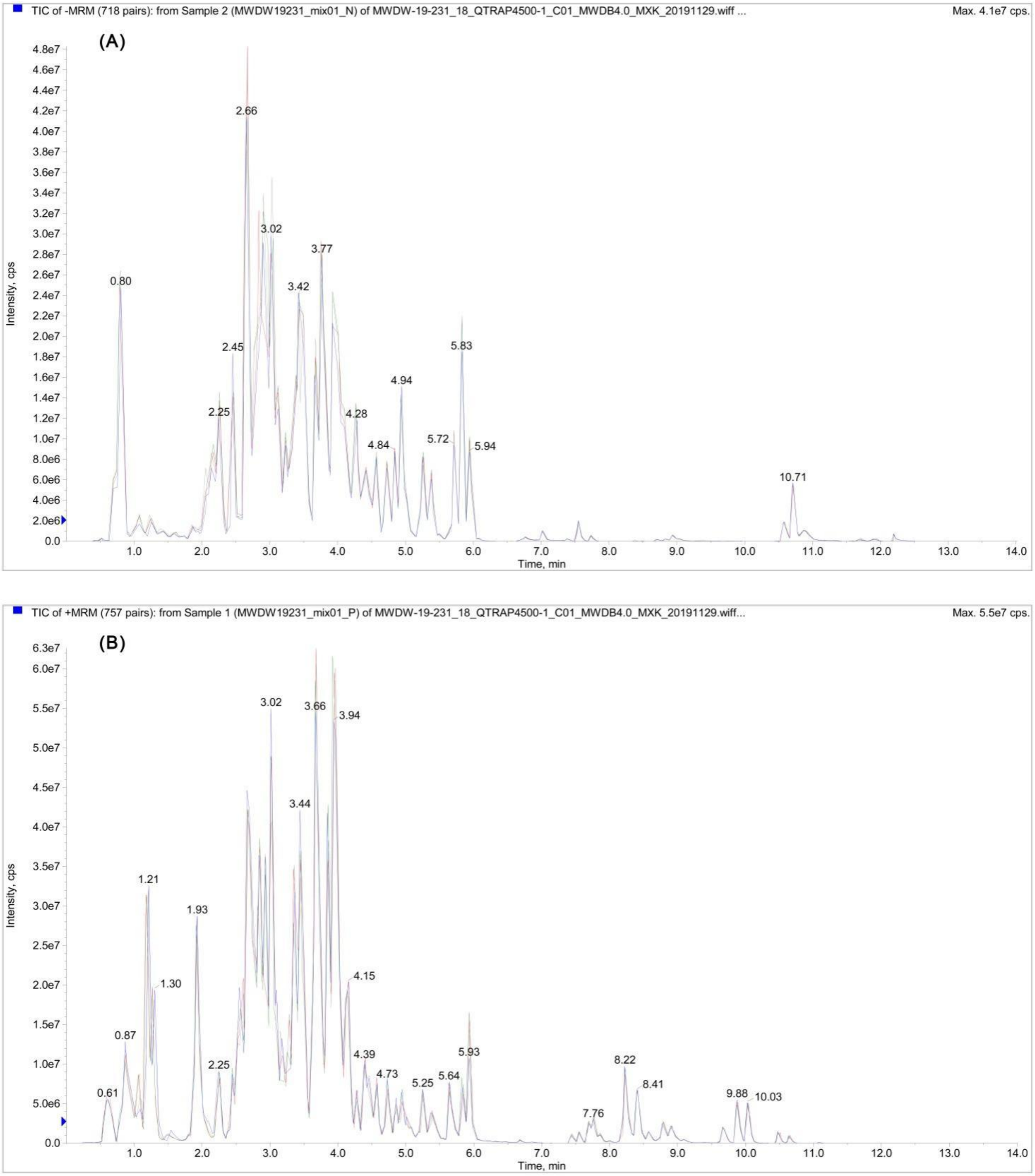

Supplementary Figure 3. Superposition of the diagram for total ions current (TIC) detected between the QC samples.

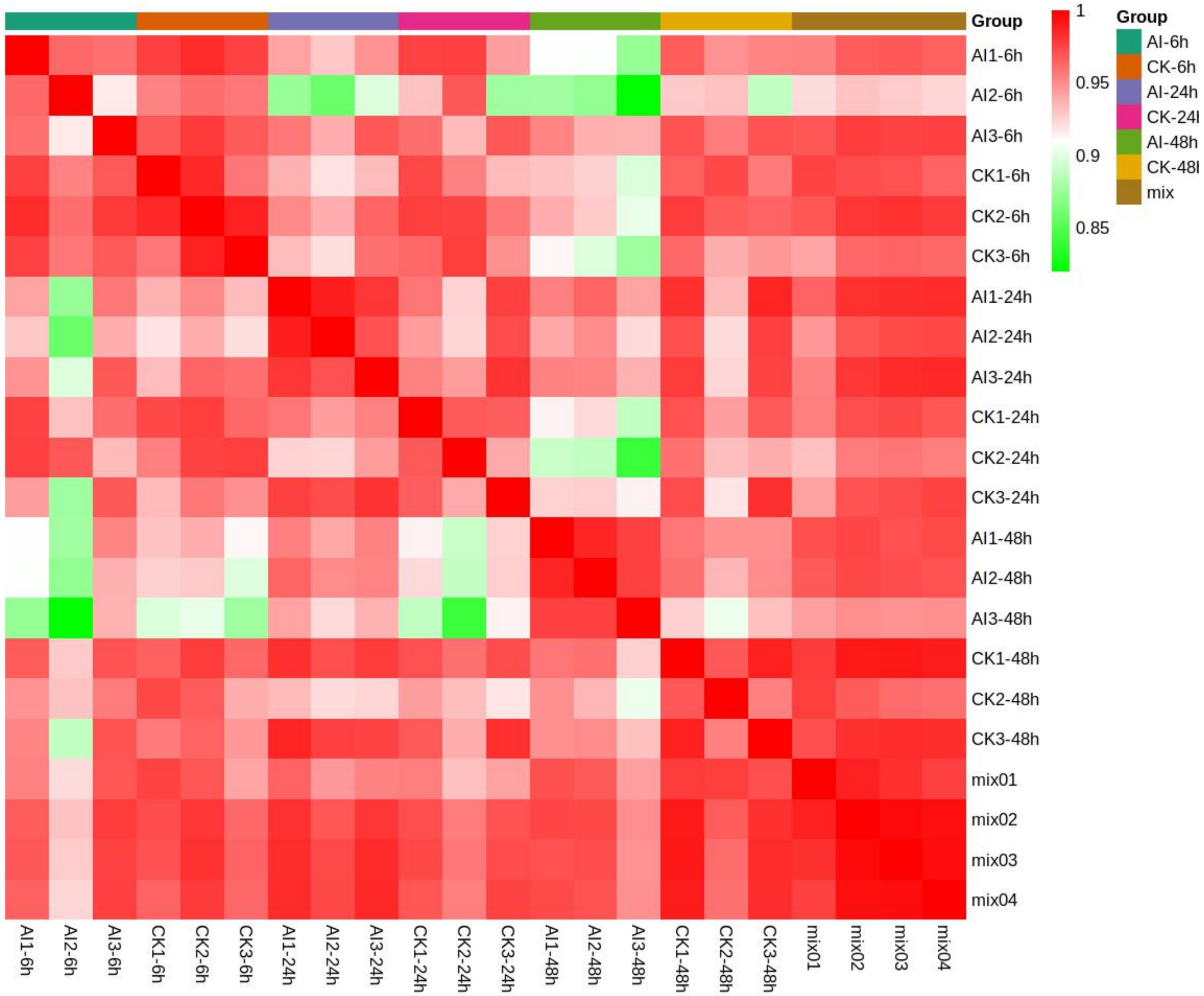

Supplementary Figure 4. Graph plotting the correlations between all the samples. The 'mix01-04' was composed of some samples randomly mixed together.

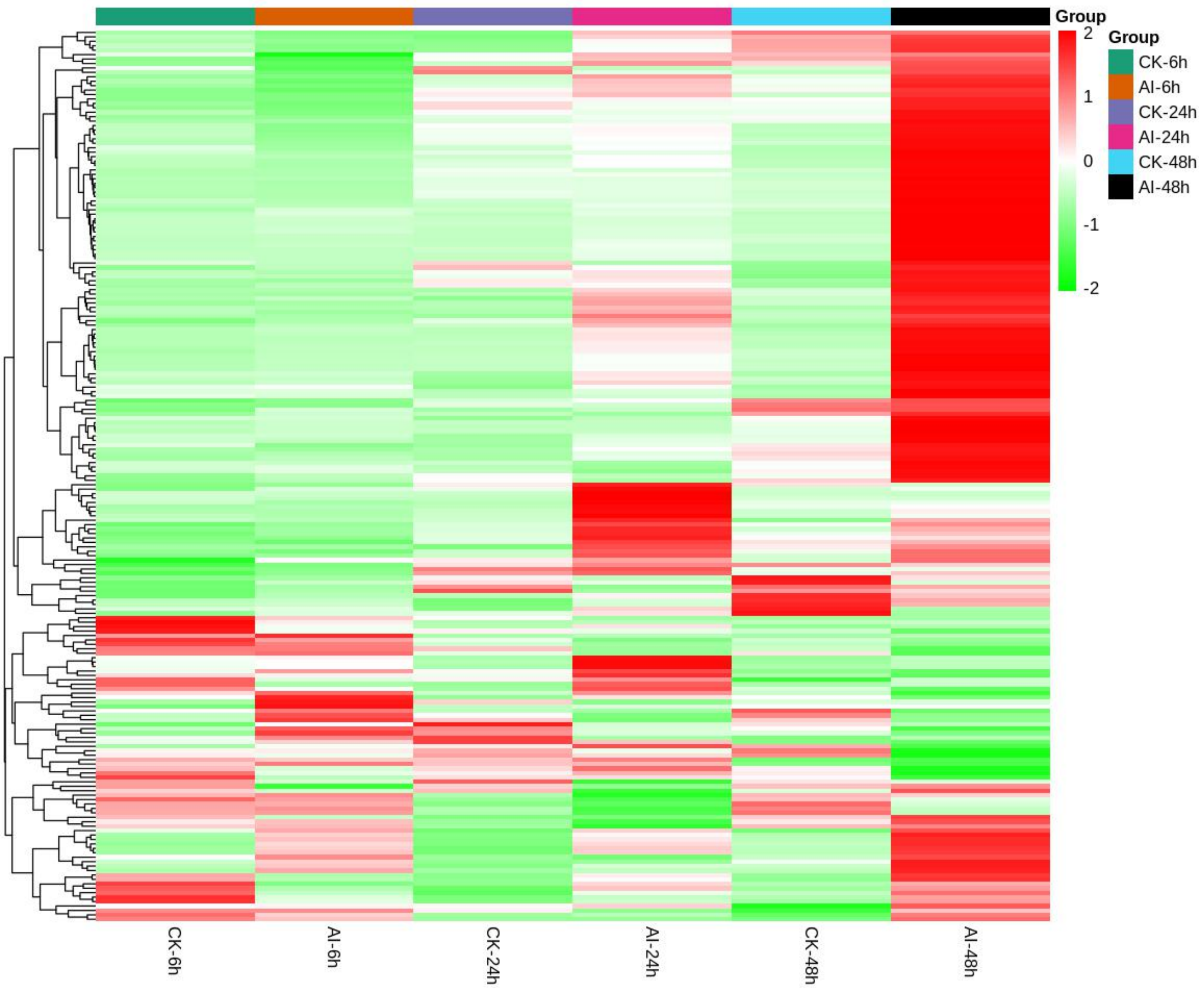

Supplementary Figure 5. Heat map of DAMs between AI and CK treatment at three stages of infection.

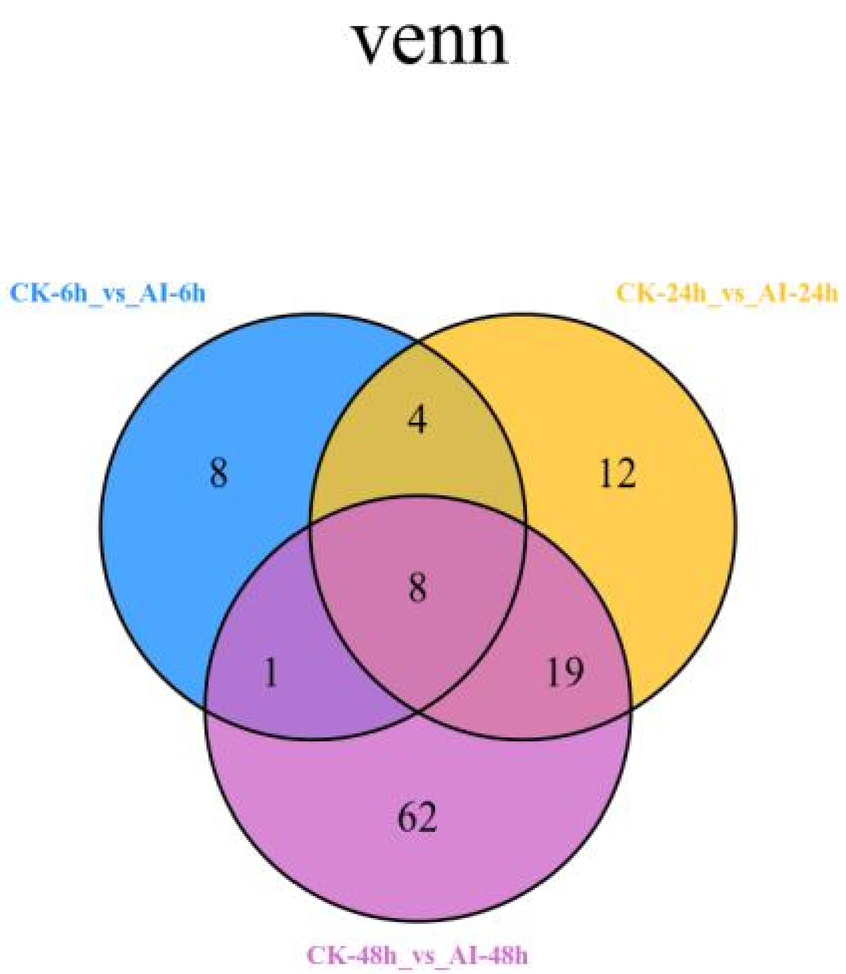

Supplementary Figure 6. Venn diagram showing the DAMs between different stages of infection.

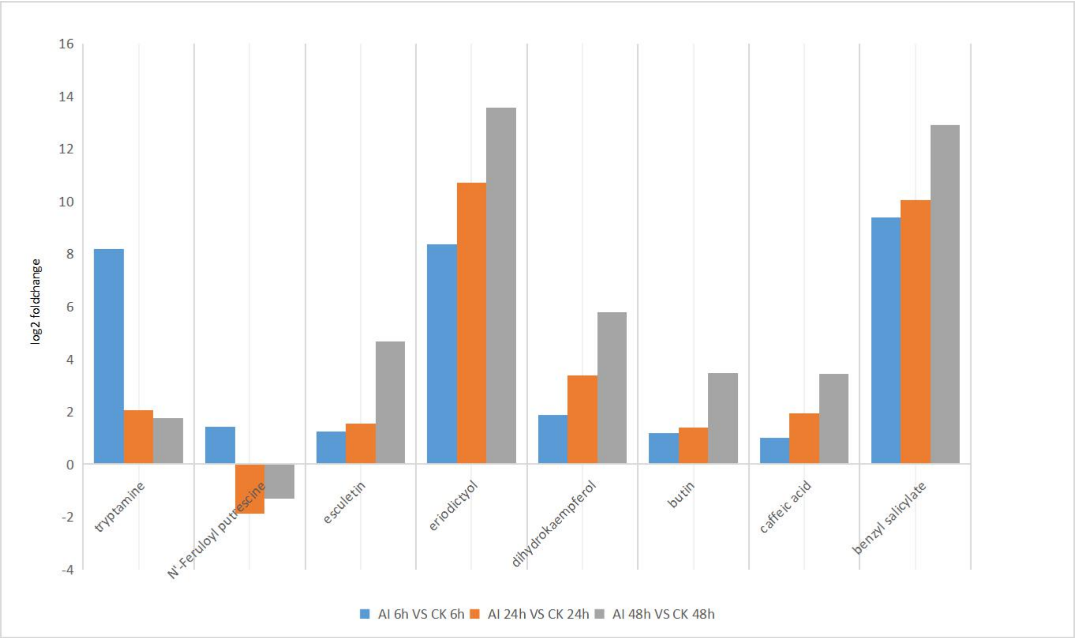

Supplementary Figure 7. The accumulation for the eight metabolites having significant differences in all the three stages.

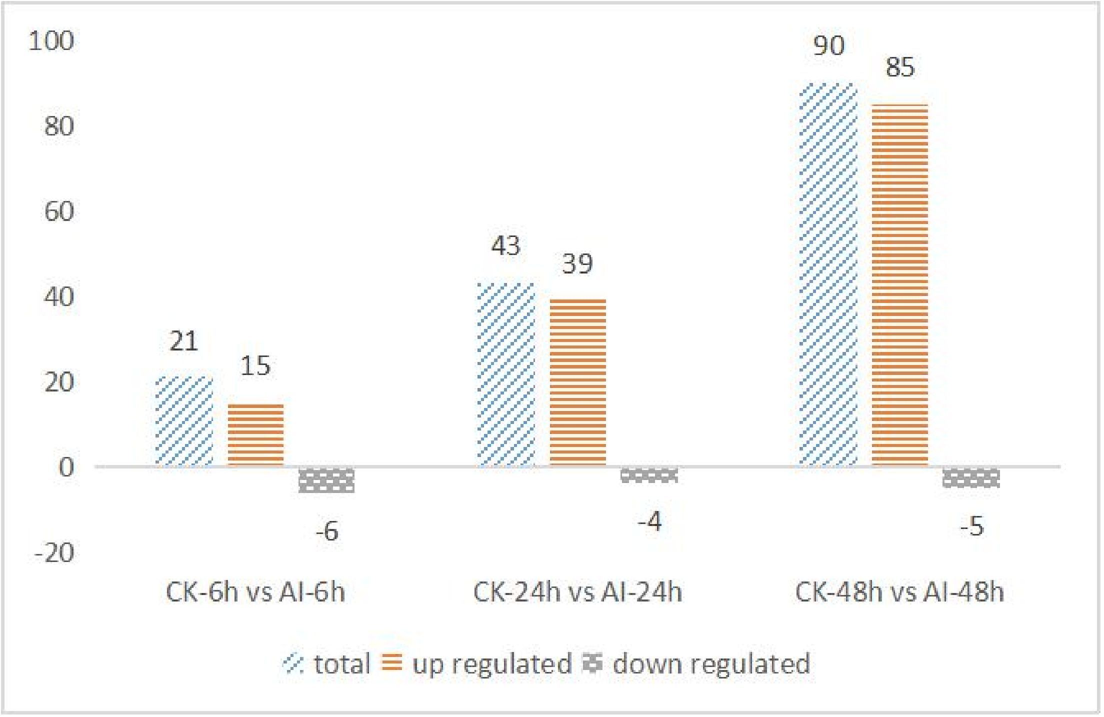

Supplementary Figure 8. The number of up-regulated and down-regulated DAMs in different stages of infection.

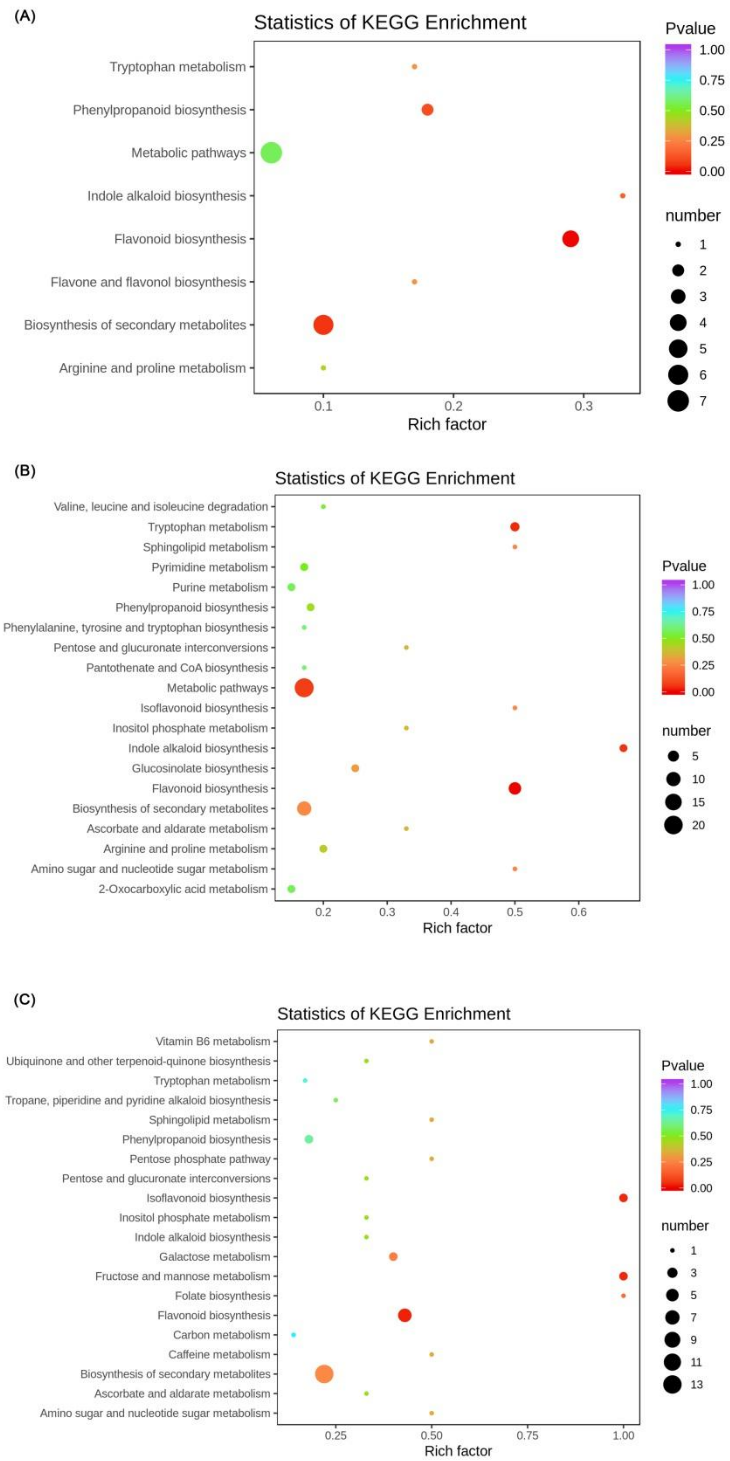

Supplementary Figure 9. Enrichment of KEGG annotations of DAMs in *Lilium* ' Sorbonne ' infected with *B. elliptica* (a) at 6 hpi, (b) at 24 hpi, and (c) at 48 hpi.

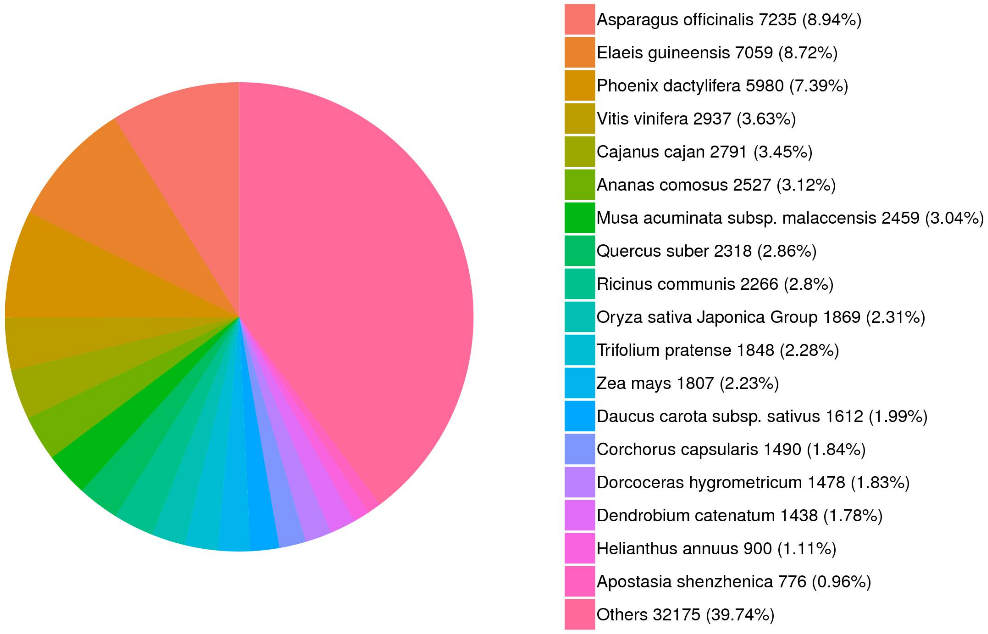

Supplementary Figure 10. The BLASTX hit species distribution in the NR database.

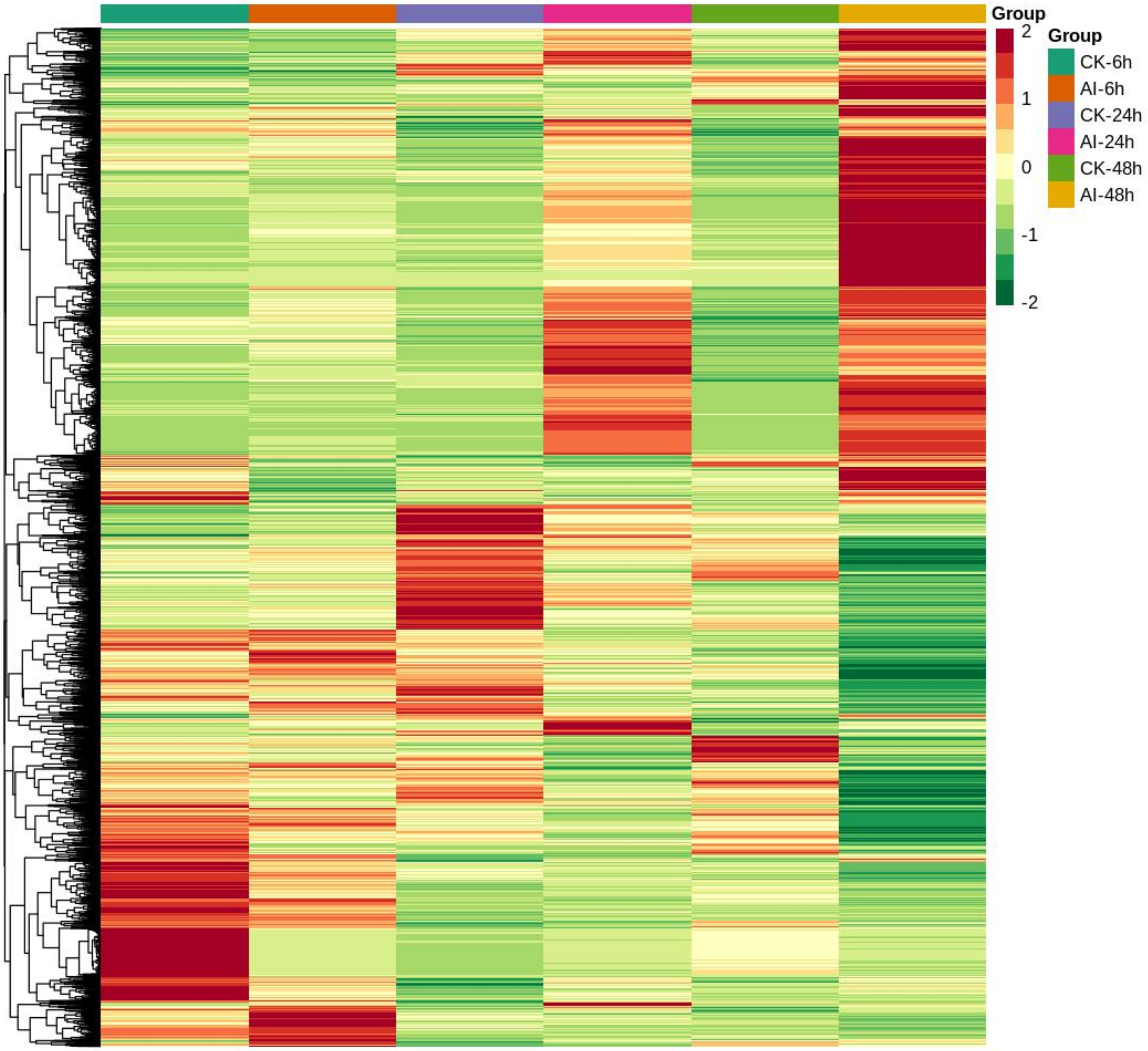

Supplementary Figure 11. Heat map of DEGs between the AI and CK treatment at three stages of infection.

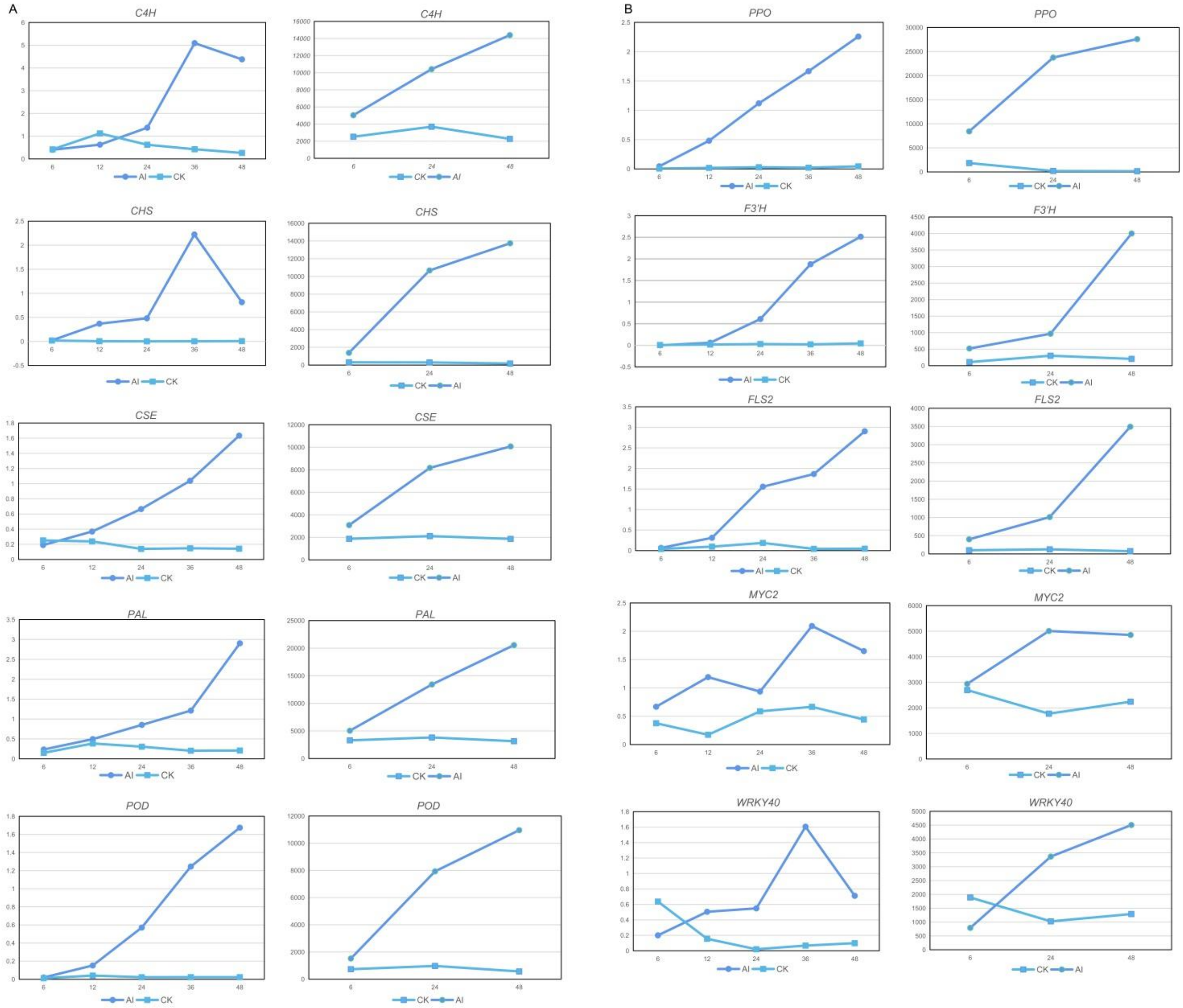

Supplementary Figure 12. The qRT-PCR of 10 plant defense-related genes. Lines with circle spots represent the gene expression levels of the leaves inoculated with *B. elliptica*. Lines with square spots the gene expression levels of the mock inoculated samples. The results of qRT-PCR were on the left, and the results of RNA-seq were on the right.

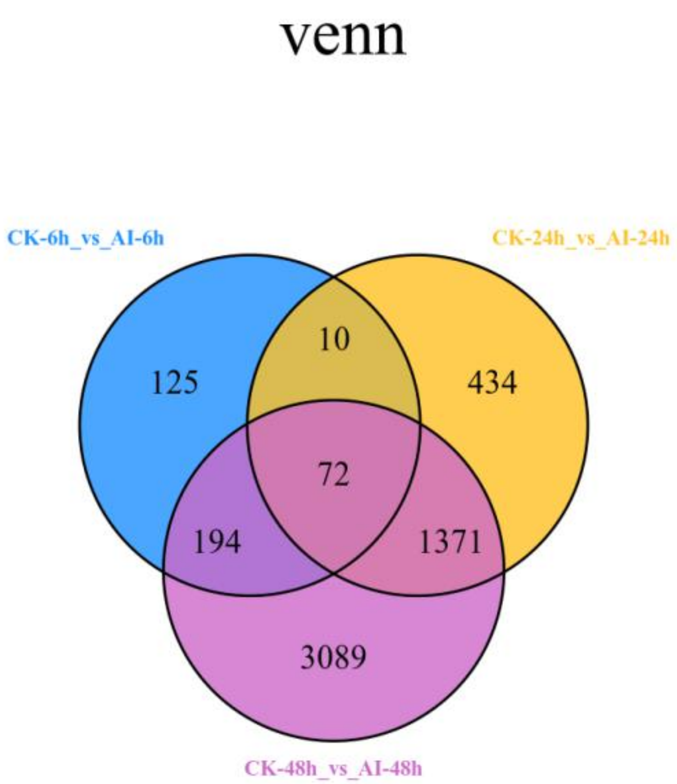

Supplementary Figure 13. Venn diagram showing the DEGs between the different infection stages.

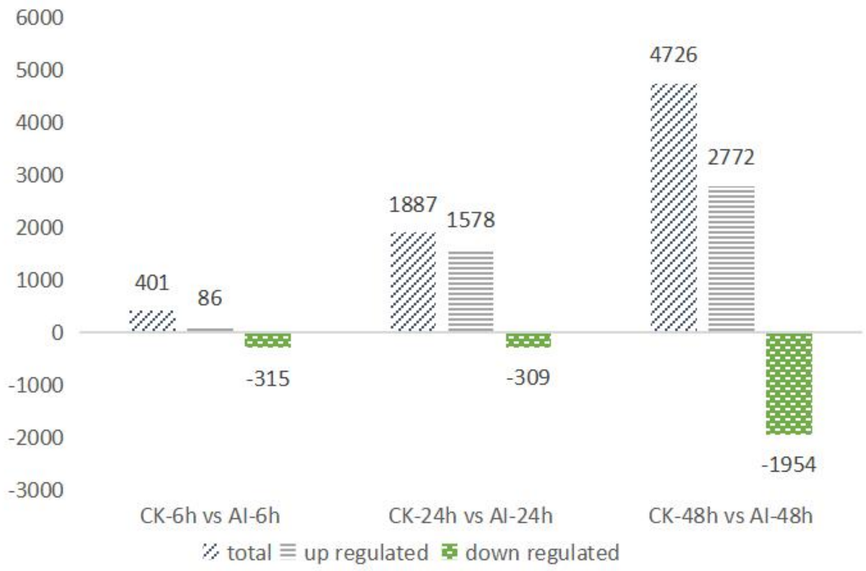

Supplementary Figure 14. The number of up-regulated and down-regulated DEGs in the different stages.

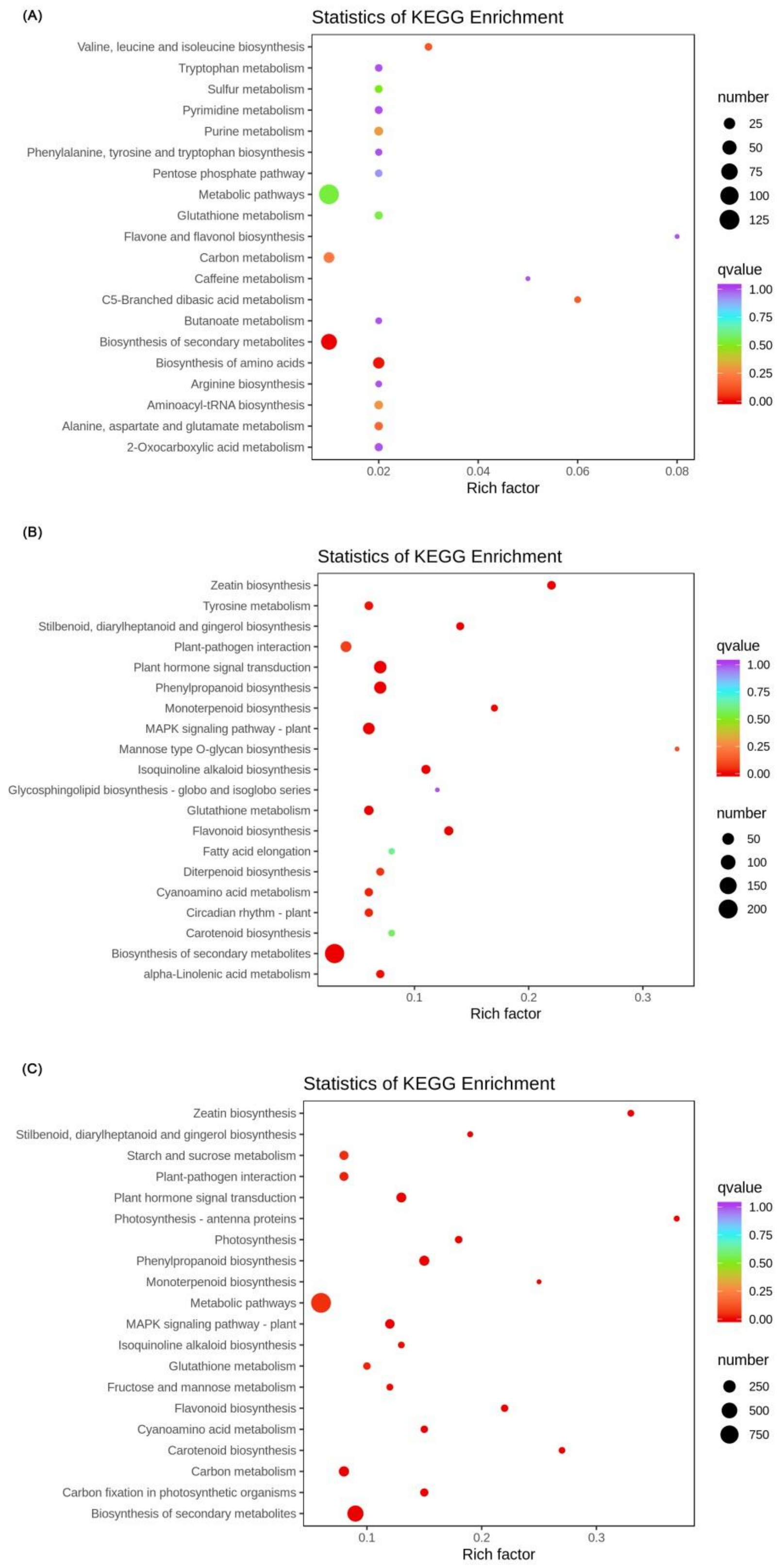

Supplementary Figure 15. Enrichment of KEGG annotations of DEGs in *Lilium* 'Sorbonne' infected with *B. elliptica* (a) at 6 hpi, (b) at 24 hpi, and (c) at 48 hpi.

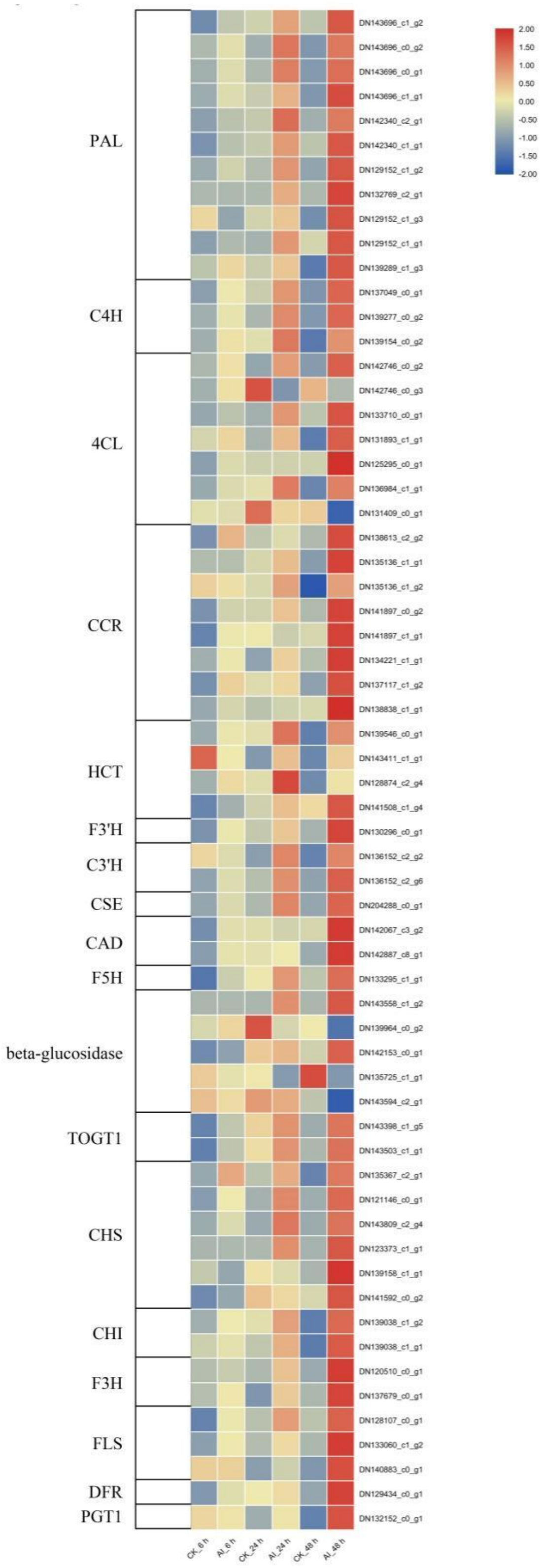

Supplementary Figure 16. The heat map of structural genes in phenylpropanoid and flavonoid Pathway.
